# Evaluating chromatographic techniques for the aroma profiling of kombucha

**DOI:** 10.1007/s00216-025-06257-5

**Published:** 2025-12-15

**Authors:** Sarah C. Foster, Laura Tipton, Katelynn A. Perrault Uptmor

**Affiliations:** 1 Nontargeted Separations Laboratory, Chemistry Department, William & Mary, 540 Landrum Drive, Williamsburg, VA 23185 USA; 2 Departments of Biology and Mathematics & Statistics, James Madison University, Harrisonburg, USA

**Keywords:** GC-MS, GCxGC-TOFMS, Time-of-flight mass spectrometry, Aroma profiling, Fermentation, Kombucha

## Abstract

Kombucha is a fermented beverage made from the addition of a symbiotic community of bacteria and yeast (SCOBY) to a sugary base tea. Despite the odors associated with kombucha, few studies have examined its chemical aroma profile. The mixture of volatile organic compounds (VOCs) from tea and its fermentation is typically examined using one-dimensional gas chromatography with time-of-flight mass spectrometry (GC-TOFMS). More complex samples benefit from the higher resolution and separation capacity of comprehensive two-dimensional gas chromatography with time-of-flight mass spectrometry (GC×GC-TOFMS), which separates analytes based on their affinity for two distinct stationary phases. This study aimed to compare the efficacy of GC-TOFMS to GC×GC-TOFMS, to use VOC data to differentiate between kombucha products, and to establish an understanding of the VOCs originating from tea versus microorganisms. Samples were taken from 12 sources including two SCOBYs, one starter tea, and eight kombucha teas and analyzed using GC-TOFMS and GC×GC-TOFMS. GC-TOFMS detected 127 analytes across 15 compound classes while GC×GC-TOFMS detected 182 analytes across 17 compound classes. The enhanced separation power of GC×GC-TOFMS allowed for the resolution of coelutions that occurred in GC-TOFMS data. Statistical techniques including principal component analysis (PCA), principal coordinate analysis (PCoA), hierarchical cluster analysis (HCA), Fisher ratio, and fold change were applied to the VOC data. Locally produced products contained simple esters and acids while commercial brands contained terpenes and aromatic compounds. This work builds on the overall aim of linking VOC data from microbes and tea with the flavor profile of fermented foods as examined by advanced analytical techniques.

**Graphical abstract:**

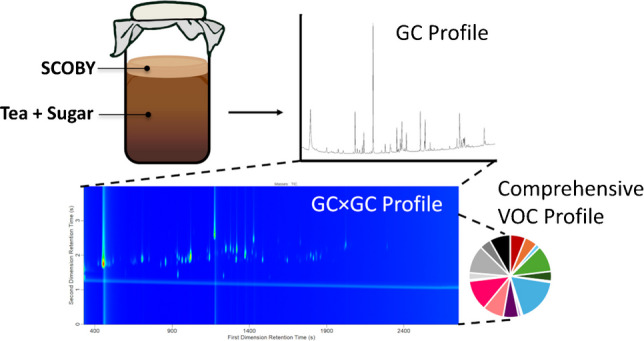

**Supplementary Information:**

The online version contains supplementary material available at 10.1007/s00216-025-06257-5.

## Introduction

Kombucha is a fermented beverage made from the addition of a symbiotic community of bacteria and yeast (SCOBY) to a sugary base tea. SCOBY microorganisms use sugar as the substrate to drive their metabolism, resulting in the release of metabolic byproducts, or volatile organic compounds (VOCs), into the tea [1]. The VOCs produced depend on the combination of bacteria and yeast used as well as the fermentation pathways followed. Kombucha fermentation occurs via several fermentation pathways including lactic acid fermentation, acetic acid fermentation, and alcoholic fermentation [2]. Typically, the fermentation process is stopped before an appreciable level of alcohol develops [3].

Kombucha is growing in popularity in the USA. Despite traditionally being brewed at home, there has been an increase in commercial breweries [4]. Federal guidelines are in place to regulate the alcohol content (% ABV) in commercially sold kombucha beverages that require brewers to label products with the total sugar content as well as the sugars added to facilitate fermentation [3, 5]. However, a study conducted by Harrison et.al. using nuclear magnetic resonance (NMR) to evaluate ethanol levels in “soft” and “hard” kombucha products (i.e., < 0.5% ethanol content differentiates soft vs. hard) determined that 12 of the “soft” kombucha products they analyzed exceeded the 0.5% ABV legal limit to be considered a non-alcoholic beverage [5]. Kombucha is receiving an increase in attention with the goal to accurately characterize its contents, which may drive changes in regulatory monitoring. The development of analytical methods for the characterization of kombucha will assist in the development of further regulatory measures for the beverage.

Fermented foods and beverages are associated with a distinct odor. Despite the strong scent associated with kombucha, there is a lack of scientific studies dedicated to understanding its chemical aroma profile. The mixture of VOCs from kombucha and its fermentation can be examined through analytical techniques such as gas chromatography-mass spectrometry (GC-MS). Traditional GC-MS separates analytes based on their chemical affinity for a stationary phase within a capillary column before sending them to a mass spectrometer for identification. Existing literature related to the VOC profiling of kombucha and tea products relies on the separation and identification capabilities of GC-MS [6–8]. However, complex samples, like kombucha, benefit from the higher resolution and separation capacity of comprehensive two-dimensional gas chromatography (GC×GC) [9]. GC×GC is becoming more common for the analysis of many foods and beverages such as beer, wine, and honey [10]. In GC×GC, separation is performed using two independent retention mechanisms. Two columns are coated with different stationary phases and separated by a modulator. Enhanced separation improves the identification of components in complex samples as it reduces uncertainty resulting from the co-elution of compounds with similar properties [11]. GC×GC is often paired with dual time-of-flight mass spectrometry (TOFMS) detection and flame ionization detection (FID) for the detection and identification of analytes [12–16]. The abilities of GC×GC-TOFMS/FID have been adapted for use on petroleum products, forensic investigations, and other fermented foods and beverages [12–16]. The FID detector is often used in VOC analysis where reverse fill/flush modulation is used since a surplus of flow is generated which cannot be sent to the TOFMS detector [17].

Profiling the volatile constituents of various teas using GC-MS has been the subject of prior studies [8, 18]. Understanding the VOCs related to teas can help inform analyses related to kombucha in order to distinguish VOCs contributed by the tea product from VOCs related to microbial metabolism. Moreover, several studies have examined kombucha during the production process including kinetic experimentation and measuring acid and alcohol levels during different stages of fermentation [3, 6]. Studies have also been conducted related to the microbial genetic composition of SCOBY [19, 20]. The majority of the literature surrounding kombucha relies on the individual production of kombucha directly for experimentation [6, 7, 21]. Further, the composition of the kombucha produced for these experiments uses either black or green tea, whereas commercial products are often a combination of both. There is limited published research available on the aroma profile of industrially produced kombucha products, and no studies have used the enhanced capabilities of GC×GC for this purpose.

This study aimed to examine commercial kombucha products and kombucha products local to the state of Virginia using gas chromatography-time-of-flight mass spectrometry and flame ionization detection (GC-TOFMS) and comprehensive two-dimensional gas chromatography with dual time-of-flight mass spectrometry and flame ionization detection (GC×GC-TOFMS). The purpose of this investigation was to compare the efficacy of GC-TOFMS to GC×GC-TOFMS, to use VOC data to differentiate between kombucha products to better understand their composition, and to establish an understanding of the VOCs composing the kombucha tea VOC contribution versus the kombucha microbial VOC contribution. Determining more about the chemical composition of commercial kombucha products will contribute to consumer understanding of product content and could potentially play a role in future regulatory efforts for kombucha production.

## Materials and methods

### Sample preparation

Twelve kombucha products were donated or purchased from local Virginia kombucha breweries and local family kitchens. Brands included Blue Ridge Bucha (Waynesboro, VA, USA), Ninja (Richmond, VA, USA), GT Foods Synergy (Beverly Hills, CA, USA), and Sage Mermaid (Norfolk, VA, USA). Multiple kombucha products were analyzed from each brand: Blue Ridge Bucha Starter Tea, Ninja Kombucha in Cranberry Lime Ginger and Goldenberry flavors, Blue Ridge Bucha in Elderberry Lime and Ginger Hibiscus (two cans of the same flavor analyzed), Sage Mermaid in Blueberry Pomegranate and Ginger Lemon, and two bottles of Synergy purchased from separate stores in Harrisonburg, Virginia and Williamsburg, Virginia. Two cans of Blue Ridge Bucha Ginger Hibiscus were analyzed because the cans were different colors and fizziness when opened. Homemade kombucha products were also donated by local families, including two SCOBYs and one batch of kombucha tea.

Three milliliters of each kombucha tea product was aliquoted into 20-mL headspace vials in biological replicates of five using a 5-mL variable volume micropipette (VWR International, Radnor, Pennsylvania, USA). Three milliliters of each SCOBY sample was aliquoted into 20-mL headspace vials in biological replicates of three due to the low volume provided. Each vial was labelled with the product identifier, replicate number, and preparation date. Approximately 0.500 g of salt was measured on an analytical balance, added to each replicate, and vortexed until dissolved to increase volatility. An empty headspace vial was used as the blank control. The kombucha products and sample vials were stored in the fridge when not running on the instrument.

An alkane standard of C_7_—C_30_ saturated alkanes (Sigma Aldrich, Saint Louis, MO, USA) was run along with each batch of kombucha samples to create linear retention indices to support analyte identification. Additionally, a cannabis terpenes standard of 19 common plant terpenes (Restek Corporation, Bellefonte, PA, USA) was run for identification confirmation of select analytes (See Electronic Supplementary Material, Table S1).

### SPME arrow sampling

Solid phase microextraction (SPME) arrow sampling with a 1.50 mm wide sleeve divinylbenzene/carbon wide range (DVB/C-WR) fiber (Restek Corporation) was performed using 20-mL headspace vials (Restek Corporation) containing 3 mL of kombucha product. This fiber was chosen due to its performance in similar studies [15, 16]. Sample extraction and injection were performed using a LECO L-PAL3 Autosampler (LECO Corporation, St Joseph, MI, USA). Sample incubation was performed for 2 min at 50 °C at 250 rpm. During incubation, sample agitation occurred for 5 s on and 2 s off. Sample extraction was performed for 10 min at 50 °C at 1000 rpm. The needle penetration depth was 40 mm into the sample vial, and the penetration speed was 20 mm/s. Injection was performed to a depth of 33 mm at 10 mm/s with a desorb time of 2 min. The SPME arrow fiber was conditioned at 270 °C for 30 min before each sequence and confirmed with a fiber blank injection. The SPME arrow fiber was reconditioned for 5 min prior to individual sample injection and for 2 min after injection. Samples were run as they were obtained to minimize storage impacts.

### GC × GC-TOFMS method

The instrument used for analysis of kombucha VOCs was a Pegasus BTX GC×GC with a Paradigm Shift™ reverse fill-flush (RFF) flow modulator and dual channel detection using a flame ionization detector (FID) and a time-of-flight mass analyzer (LECO Corporation). The carrier gas was ultra-high purity helium (Airgas, Radnor, PA, USA) at a flow rate of 0.5 mL/min. The first-dimension column (^1^*D*) was an Rxi-5MS column (20.0 m × 0.18 mm ID × 0.18 µm *d*_f_, Restek Corporation). The second-dimension column (^2^*D*) was a Rxi-17Sil MS (3.7 m × 0.25 mm ID × 0.25 µm *d*_f_, Restek Corporation). The ^1^*D* flow rate was 0.5 mL/min, and the ^2^*D* flow rate was 30 mL/min. The sample loop dimensions were 0.17 m × 0.53 mm ID resulting in a loop volume of 38 µL. The modulation period was 4 s, and the flush time was 160 ms throughout the duration of the run. This resulted in a flush factor of 1.76 which exceeds the 1.5 times fill volume recommended for full transfer. The calculated flow to the TOFMS was 1.03 mL/min, and the calculated flow to the FID was 37.18 mL/min. The inlet was operated in splitless mode for better detection of low-level analytes. The septum purge flow was 3 mL/min, and the inlet purge time was 30 s with a purge flow of 20 mL/min and a total inlet flow of 50.5 mL/min. The initial temperature for the GC oven was 40 °C which was held for 2 min, and then the oven was ramped at 5 °C/min until a target temperature of 250 °C was reached, with a final hold of 2 min, resulting in a runtime of 46 min. The transfer line was held at 345 °C, and the ion source temperature was 300 °C. The TOFMS operated via electron impact (EI) ionization resulting in an acquisition rate of 100 scans/s and an extraction frequency of 30 kHz for the mass range of 30–550 *m/z*. The FID was set to 345 °C and operated at 100 Hz. The flow rate for hydrogen (ultra-high purity, Airgas) fuel was 45 mL/min. The flow rate for air (ultra zero purity, Airgas) was 450 mL/min. The flow rate for the nitrogen (ultra-high purity, Airgas) makeup gas was 25 mL/min. Data acquisition was performed for both detectors using LECO ChromaTOF software V5.59.02 with data processing V1.2.0.6 (LECO Corporation). Both the FID and TOFMS had an acquisition delay of 330 s to mitigate matrix signal. GC-TOFMS and GC×GC-TOFMS data were exported as.SMP files to a workstation computer with the same ChromaTOF processing software. Collected FID data were not included in the final analysis.

### GC-TOFMS method

A subset of the kombucha samples were run in 1D for comparative purposes. The instrument used for analysis of kombucha VOCs was a Pegasus BTX GC×GC with a Paradigm Shift™ reverse fill-flush (RFF) flow modulator and dual channel detection using a flame ionization detector (FID) and a time-of-flight mass analyzer (LECO Corporation); however, for 1D analysis, the instrument was operated in 1D mode by disabling the modulator settings. The column was an Rxi-5MS column (20.0 m × 0.18 mm ID × 0.18 µm *d*_f_, Restek Corporation). Ultra-high purity helium (Airgas) was used as the carrier gas with a flow rate of 0.5 mL/min. The initial temperature for the GC oven was 40 °C which was held for 2 min, and then the oven was ramped at 5 °C/min until a target temperature of 250 °C was reached, with a final hold of 2 min, resulting in a runtime of 46 min. The transfer line was held at 345 °C, and the ion source temperature was 300 °C. The TOFMS operated via electron impact (EI) ionization resulting in an acquisition rate of 100 scans/s and an extraction frequency of 30 kHz for the mass range of 30–550 m/z. The FID was set to 345 °C and operated at 20 Hz. The flow rate for hydrogen (ultra-high purity, Airgas) fuel was 45 mL/min. The flow rate for air (ultra zero purity, Airgas) was 450 mL/min. The flow rate for nitrogen (ultra-high purity, Airgas) makeup gas was 25 mL/min. Data acquisition was performed for both detectors using LECO ChromaTOF software V5.59.02 with data processing V1.2.0.6 (LECO Corporation). Both the FID and TOFMS had an acquisition delay of 330 s to mitigate matrix signal. GC-TOFMS and GC×GC-TOFMS data were exported as.SMP files to a workstation computer with the same ChromaTOF processing software. Collected FID data were not included in the final analysis.

### Data processing method

Samples were exported as.SMP files after the chromatographic run to a Network Attached Storage (NAS) system. Files were downloaded from the NAS to an offline data workstation and imported into a ChromaTOF database V5.59.02 with data processor V1.2.0.6 (LECO Corporation). Mass spectra were searched in the NIST Mass Spectral Library Version 3.0, 2023, using the Mainlib and Replib components of the library. Each alkane standard was processed with a basic peak finding method to identify the alkanes and added to separate linear retention index (RI) methods based on sample batches by adding the identified alkane’s retention time to a retention index table and multiplying the carbon number by 100.

For the data processing method within ChromaTOF, peak finding was enabled using the XIC quantitation signal and a mass tolerance of 0.3 Da. The minimum S/N was 300 to maximize the identification of low-level analytes. Library searching was enabled. The minimum stick count was 3, the mass range for library searching was 30–550 *m/z*, the relative abundance was 10, the minimum similarity for a match was 500, and the minimum similarity before a hit was assigned was 700. Retention index was enabled. An alkane standard was run with each batch of samples, and retention indices were calculated based on the alkane standard in the respective batch. After processing each chromatogram, column bleed was removed, and identifications were confirmed manually by examining mass spectra and NIST retention index data. Compounds were given an identity if the retention index value in ChromaTOF was within the range of retention index values provided in the NIST library for a semi-standard nonpolar column. The range of recorded values in NIST was denoted by a ± symbol. 1D data were processed using the same method, except decreasing the *S*/*N* to 10 to account for the lower acquisition rate.

To supplement peak finding with statistical processing, ChromaTOF Tile® v1.3.50.0 (LECO Corporation) and ChromaTOF Sync2D® v2.0.15.0 (LECO Corporation) were used. ChromaTOF Tile reviews raw GC×GC data to identify differences between samples or groups of samples based on class averages. ChromaTOF Sync2D performs peak deconvolution and peak alignment to generate a single peak table with aligned analyte retention times for an entire sample set.

Within ChromaTOF Tile, different statistical tests can be performed. Fisher ratio and fold change tests were performed on the kombucha products to compare the VOCs found across brands and between different flavors. The Fisher ratio is a measure of class-to-class variation divided by intraclass variation used to identify class-distinguishing differences. Fold change is a measure of large relative change between samples and can be calculated for individual samples or between two groups of samples. The common parameters used for both statistical tests were a 1D tile size of 4 and a 2D tile size of 26 (auto-calculated in the software based on the height and width of a peak in ChromaTOF), a *S*/*N* threshold of 300, a total of 1 sample must exceed *S*/*N* threshold, 1 mass *F*-ratio to average, a minimum of 3 masses per tile, a minimum mass of 30 *m/z*, and a maximum mass of 550 *m/z*. For analysis, two approaches were used. In the first workflow, a tile processing method was created using a Fisher ratio of 20 as the statistical threshold applied. For the second workflow, a tile processing method was created using a fold change value of 1 as the statistical threshold applied. After processing the data, a list of “hits” was generated which included either the *F*-ratio value or the fold change, alongside the mean retention times in the first and second dimension, the quant mass, and a heatmap of relative amounts in each sample class average for each hit. Hits were accepted or rejected based on comparison of the retention times and quant mass in ChromaTOF. Once a list of chemical features was compiled, identifications were confirmed by comparing sample mass spectra to NIST library mass spectra as well as comparing experimental retention index values to those in the NIST library. For each statistical test performed in ChromaTOF Tile, PCA scores and loadings plots were generated using logarithmic scaling and mean centering.

For the ChromaTOF Sync2D processing method, the expected full-width-half-height (FWHH) was 0.05, the *S*/*N* was 100, the expected 1D retention shift was 4 s, and the expected 2D retention shift was 0.05 s. Expected retention shift was calculated by subtracting the lowest 1D or 2D retention time from the highest 1D or 2D retention time for a single peak. The modulation period was extracted from the uploaded data files, downsampling sums were set to none, and no masses were excluded. Library searching parameters were kept the same as in ChromaTOF. Statistical processing was performed after initial processing and manual feature filtering. Features were accepted if the quant mass identified was present at the same retention time in ChromaTOF Sync2D and ChromaTOF. Features were deleted if the quant mass identified was not present at the same retention time in ChromaTOF Sync2D and ChromaTOF or if it was determined that the quant mass identified was a fragment from a nearby feature or peak. The identification of the peak was confirmed by comparing sample mass spectra to NIST library mass spectra, as well as an RI match within 10–15 of the NIST library experimental RI data. The feature list generated by ChromaTOF Sync2D was supplemented by various ChromaTOF Tile analyses. Due to the different processing methods between ChromaTOF Tile and ChromaTOF Sync2D, some features were identified in ChromaTOF Tile but not in ChromaTOF Sync2D. If a feature was identified by ChromaTOF Tile and not in ChromaTOF Sync2D, it was added to the peak table in ChromaTOF Sync2D by specifying the retention times and specific quant mass identified in ChromaTOF Tile. For each statistical test performed in ChromaTOF Sync2D, PCA scores and loadings plots were generated using logarithmic scaling and mean centering, and *p*-values were calculated for individual features.

GC-TOFMS data were processed in ChromaTOF Sync2D operated in 1D alignment mode. The FWHH value was 0.2, the *S*/*N* was 10, and the expected maximum retention time shift was 5 s. Library searching parameters were kept the same as in ChromaTOF. Statistical processing was performed after initial processing and manual feature filtering. The identification of the peak was confirmed by comparing sample mass spectra to NIST library mass spectra, as well as comparing the retention time and mass spectra to the data collected in 2D as confirmed by retention indices. For each alignment performed in ChromaTOF Sync2D, PCA scores and loadings plots were generated using logarithmic scaling and mean centering, and *p*-values were calculated for individual features.

Sync2D data were exported as a.CSV file for further ordination in R statistical software [22]. Heatmapping and hierarchical cluster analysis (HCA) were performed on the entire data set (all analytes detected in all samples along with detected peak areas) using the ComplexHeatmap package [23]. Principal component analysis (PCA) and principal coordinate analysis (PCoA) were performed as well using the *vegan* package [24].

Feature selection for each brand was performed in ChromaTOF using mass spectral data to identify the peaks present in the samples in 1D. Feature selection for each brand was performed using Sync2D area charts to visually determine the samples in which an analyte was present. For both 1D and 2D feature selection, the analyte was required to have a peak area surpassing that of the background in the blank samples in a majority of replicates.

## Results and discussion

### Comparison of GC-TOFMS and GC × GC-TOFMS

**Fig. 1:**
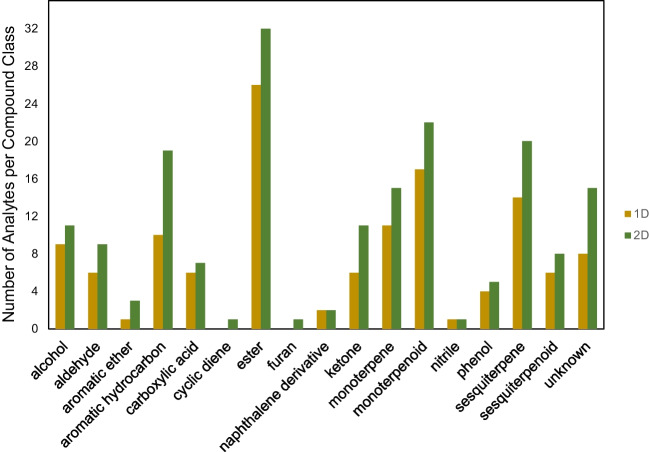
Bar chart comparing the number of analytes detected across compound classes using GC-TOFMS (1D) and GC×GC-TOFMS (2D)

After data processing, 127 analytes belonging to 15 different compound classes were identified across 13 distinct sample groups using GC-TOFMS (1D GC). A total of 182 analytes belonging to 17 different compound classes were identified across the same sample groups using GC×GC-TOFMS. The compound classes detected using 1D GC included alcohols, aldehydes, aromatic hydrocarbons, carboxylic acids, esters, naphthalene derivatives, ketones, monoterpenes, monoterpenoids, nitriles, phenols, phenylpropanoids, sesquiterpenes, and sesquiterpenoids. In addition to the previously listed classes, GC×GC detected aromatic ethers, cyclic dienes, and furans. GC×GC resulted in a greater number of analytes found in each compound class, not including naphthalene derivatives, nitriles, and phenylpropanoids which were detected at equal numbers using both techniques, as shown in Fig. 1. A complete list of analytes detected using 1D GC and GC×GC can be found in Table S1 in the Electronic Supplementary Material.

**Fig. 2:**
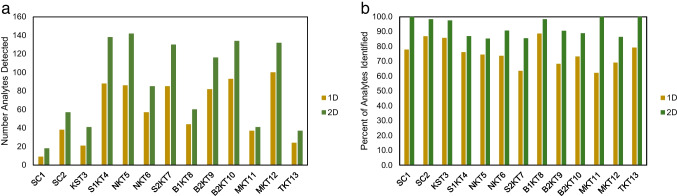
**a** Bar chart comparing the number of analytes detected in each sample group using GC-TOFMS (1D) and GC×GC-TOFMS (2D) and **b** bar chart comparing the percentage of analytes detected that could be given an identity out of the total number of analytes identified in each brand using GC-TOFMS (1D) and GC×GC-TOFMS (2D). Sample groups shown include SCOBY 1 (SC1), SCOBY 2 (SC2), Blue Ridge Bucha Starter Tea (KST3), Synergy 1 (S1KT4), Ninja Kombucha Cranberry Ginger Lime (NKT5), Ninja Kombucha Goldenberry (NKT6), Synergy 2 (S2KT7), Blue Ridge Bucha Elderberry Lime (B1KT8), Blue Ridge Bucha Ginger Hibiscus 1 (B2KT9), Blue Ridge Bucha Ginger Hibiscus 2 (B2KT10), Sage Mermaid Blueberry Pomegranate (MKT11), Sage Mermaid Ginger Lemon (MKT12), and Family tea (TKT13)

Studies looking at fermented black tea products using GC-MS have recorded anywhere from 81 VOCs up to as many as 764 volatile compounds depending on the experimental setup and aim of the study [8, 18]. Despite the range in the number of analytes detected, the general compound classes found in studies looking at fermented teas are consistent with the classes found in this study [6]. However, the inconsistency across literature of sample preparation and instrumentation as well as the number of analytes found using GC-MS provided challenges when comparing the experimental results to previous studies. Approximately twice as many analytes were found in each sample group using GC×GC as compared to 1D GC. Moreover, a greater percentage of the analytes detected in each sample group were able to be identified with mass spectral searching using the GC×GC system, possibly due to greater separation providing more distinct mass spectra for the selected peaks (Fig. 2).

**Fig. 3:**
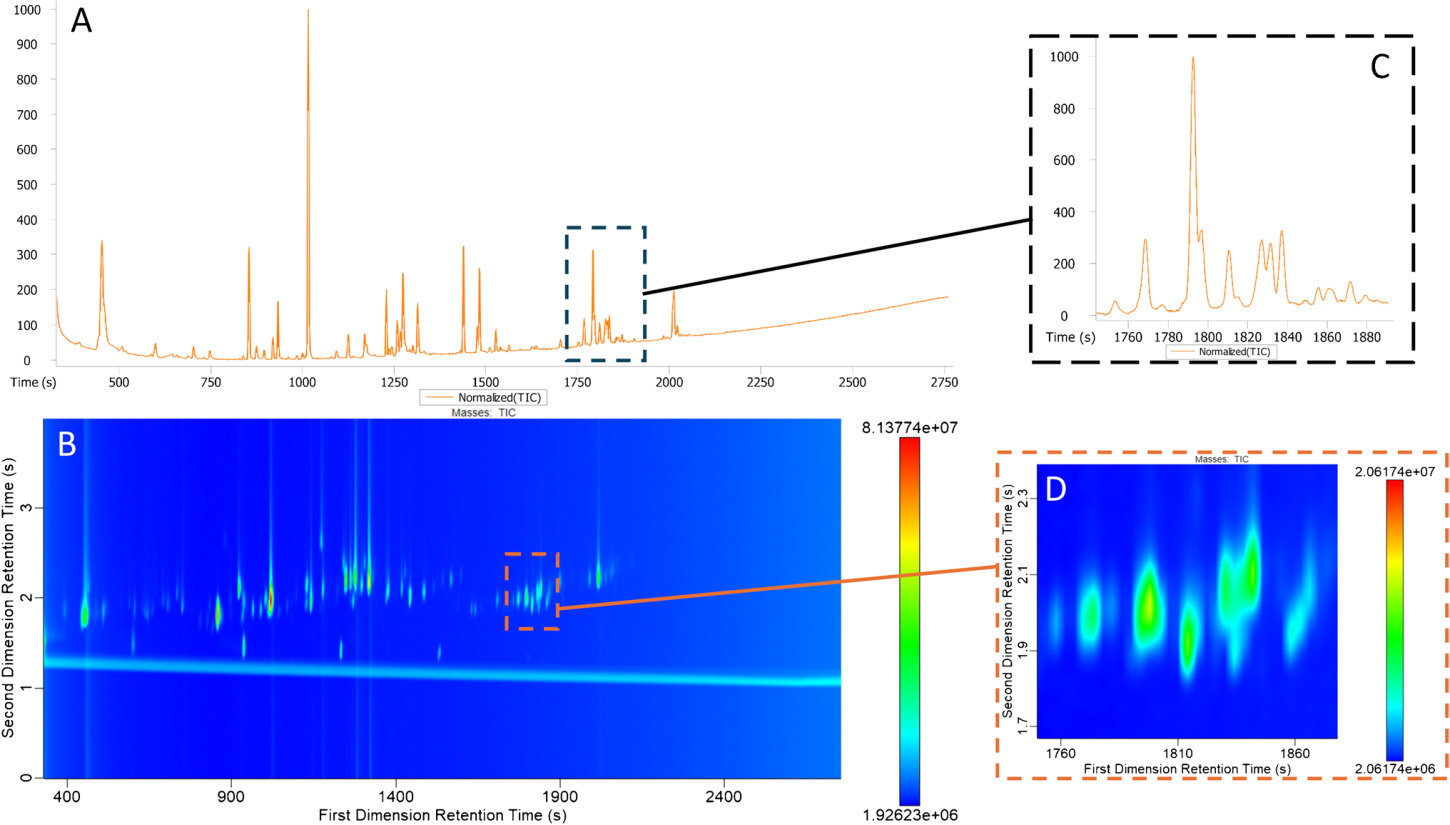
Comparison of **A** a one-dimensional total ion current chromatogram of Synergy kombucha (S1KT7-1) and **B** a two-dimensional total ion current chromatogram of Synergy kombucha (S1KT7-1). **C** shows a close-up image of the co-elution in the 1D chromatogram from 1750 to 1880 s, and **D** shows a close-up image of the same peaks in the 2D chromatogram from 1750 to 1880 s

As seen in Fig. 3, the chromatograms generated using 1D GC and GC×GC both demonstrate good resolution and peak shape. However, areas of challenging coelutions in the 1D chromatogram, such as from 1230 to 1320 s and 1760 to 1850 s, were better resolved in the 2D chromatogram. Analytes such as 2-methylpropyl hexanoate, ethyl benzoate, and 1-ethenyl-4-methoxybenzene which eluted from 1234 to 1270 s were not seen in the 1D chromatogram. Similarly, 2-methyl-acetophenone and dimethylbenzeneethanol had the same primary retention time as octanoic acid, 1270 s. In the 1D chromatogram, only octanoic acid was detected, demonstrating the benefits of two-dimensional GC in resolving complex peak coelutions that could go undetected in one-dimensional GC. Analytes in the 1760 to 1850 s region of the 2D chromatogram, while more distinct than in the 1D chromatogram, are still slightly coeluted. Coelution in the later parts of the chromatogram could be solved with further method optimization. However, optimizing in order to separate analytes later in the chromatographic run could compromise the separation achieved at the beginning of the run. Introducing oven holds in the middle of the run can also introduce peak broadening and prevent the ability to use retention indices for identification. It is of note that all peaks detected from 1760 to 1850 s could be assigned an identity in both the 1D chromatogram and 2D chromatogram. Still, it is likely that the peak areas found using GC×GC are more accurate than the peak areas found using 1D GC due to improved chromatographic resolution of compounds.

Overall, the greater number of analytes detected using 2D GC due to the resolution of co-eluting compounds provides evidence for the implementation of comprehensive two-dimensional gas chromatography for the analysis of fermented beverages. While 1D GC was effective in separating a large number of analytes, many compounds responsible for flavor, like esters and ketones, were found in greater numbers using multidimensional chromatographic techniques.

### Understanding the volatile composition of kombucha products

**Fig. 4:**
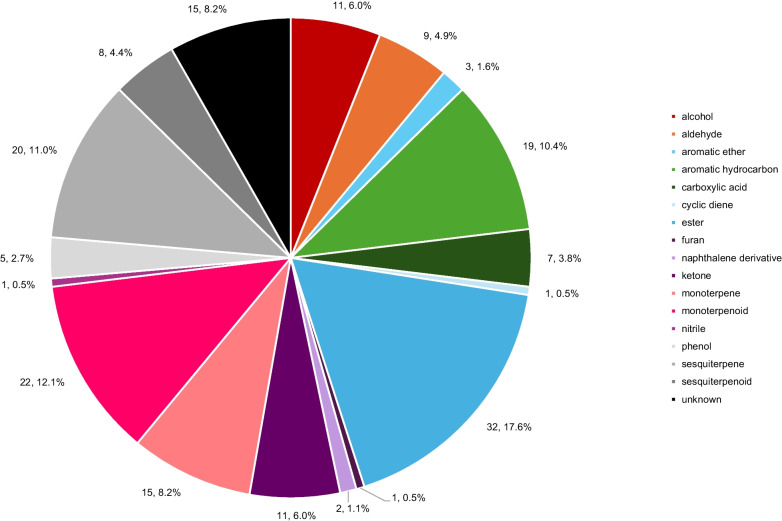
Pie chart describing the compound class composition of the analytes detected using GC×GC-TOFMS. The first number in each label describes the count of the total analytes detected, and the second number represents the percentage contribution of each compound class to the overall total of detected analytes

Results discussed herein are the results of the GC×GC analysis. Because all the analytes detected in 1D were also found in the 2D analysis, along with additional analytes, the following objectives were examined using GC×GC data rather than 1D GC data. References to corresponding one-dimensional chromatographic analyses can be found in the Electronic Supplementary Material as cited in the text. All analytes given identifications were confirmed using linear retention indices (RI). A full list of analytes and their presence in each sample group can be found in the Electronic Supplementary Material, Table S2. Analytes were grouped by class as seen in Fig. 4 to observe the range of analytes present in the overall dataset.

The prevalence of esters, compounds known to have floral and fruity aromas, is consistent with the products, mainly fruit-juice enhanced teas, being examined [8]. The fermentation process is also known to produce higher esters which could contribute to the products’ relatively high ester content [6]. Terpenes, terpenoids, sesquiterpenes, and phenols are known plant products recorded in black tea analysis [3]. Aromatic ethers have been identified in vinegar as a result of acetic acid fermentation, which occurs during kombucha production [25]. The alcohols, ketones, aldehydes, and carboxylic acids present are also consistent with the fermentation process but could occur naturally in tea. The aromatic hydrocarbons largely consisted of benzene derivatives; however, there was one polycyclic aromatic hydrocarbon (PAH) recorded, x,x-dimethylnaphthalene. PAHs are commonly found on tea leaves due to the heating that occurs during the treatment process, so the detection of a PAH is logical within the scope of the study [26]. Other naphthalene derivatives have been recorded previously in *Aloe barbadensis* [25]. This is a common ingredient in food products and beverages [27]. While none of the products analyzed clearly indicated *Aloe barbadensis* as an ingredient, it is reasonable to suspect naphthalene derivatives were naturally present in the kombucha tea since they are present in other plant samples, such as *A. barbadensis*, and are unlikely to be a result of contamination from the analysis. Cyclic dienes, furans, and nitriles have all been documented as natural food or beverage aroma contributors [3, 28–30].

Comparing the VOCs detected across all 13 products, only 6 analytes were found in every sample group: linalool oxide, 2-methylpropyl 3-hydroxy-2,2,4-trimethylpentanoate, butyl acetate, ethylbenzene, hexanoic acid, and *p*-xylene. The analytes found in all 13 sample groups all have links in literature to tea or tea-based products. Linalool oxide has been detected in multiple studies; notably, it was found in plain black tea as well as tea with added aromatics and black tea mixed with green tea [18]. 2-Methylpropyl 3-hydroxy-2,2,4-trimethylpentanoate has been found in green tea extracts [31]. Butyl acetate was found in fermented tea blended with rose hip tea, lemongrass tea, lavender tea, and peppermint tea [32]. Ethylbenzene, hexanoic acid, and *p*-xylene were identified in kombucha in an aroma study across fermentation time [7].

**Fig. 5:**
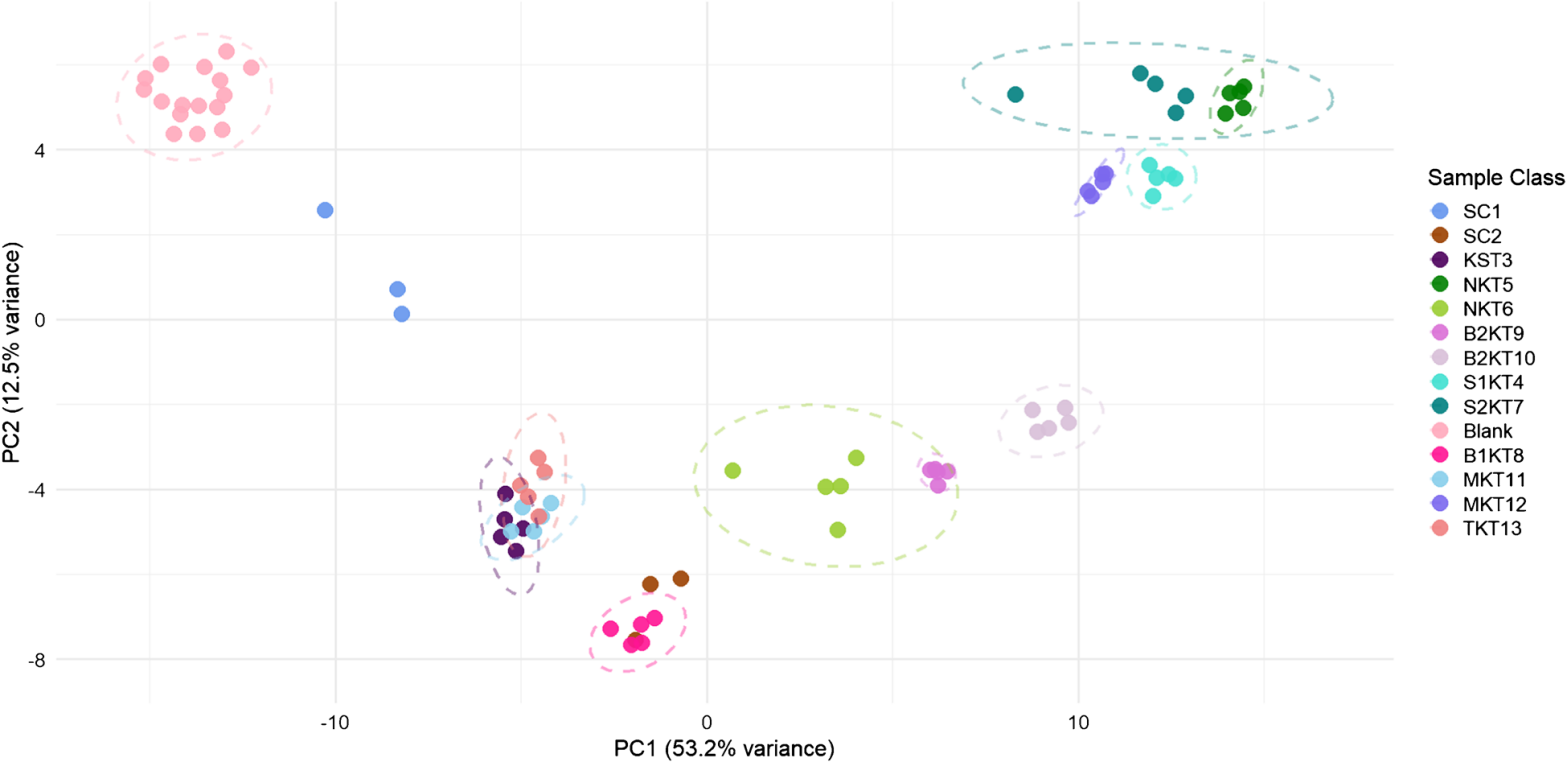
Principal component analysis (PCA) scores plot with logarithmic transform, mean centering, and 95% confidence ellipses for collected 2D chromatographic data. Sample groups shown include SCOBY 1 (SC1), SCOBY 2 (SC2), Blue Ridge Bucha Starter Tea (KST3), Synergy 1 (S1KT4), Ninja Kombucha Cranberry Ginger Lime (NKT5), Ninja Kombucha Goldenberry (NKT6), Synergy 2 (S2KT7), Blue Ridge Bucha Elderberry Lime (B1KT8), Blue Ridge Bucha Ginger Hibiscus 1 (B2KT9), Blue Ridge Bucha Ginger Hibiscus 2 (B2KT10), Sage Mermaid Blueberry Pomegranate (MKT11), Sage Mermaid Ginger Lemon (MKT12), Family tea (TKT13), and Blanks

In order to understand the similarities and differences between the sampled kombucha products, a common multivariate data processing technique, principal component analysis (PCA), was used (Fig. 5). PCA is an unsupervised method that allows visualization of multidimensional data on a two-dimensional set of axes and is therefore prevalent in many GC×GC studies [33]. Confidence ellipses were generated for all sample groups with five replicates. Confidence ellipses could not be generated for samples with only three replicates. SCOBY 1 was grouped near the blanks indicating a lack of VOCs which was confirmed with only 18 peaks detected in the 2D GC chromatogram. The lack of clustering of the SCOBY 1 replicates could be due to the biological variation or sample preparation from pipetting non-homogenous substances. The starter tea was grouped with the Family kombucha and Sage Mermaid Blueberry Pomegranate kombucha with a strong overlap of the confidence ellipses indicating many similarities in the VOC profile of the three products. SCOBY 2 shared a similar volatile profile with Blue Ridge Bucha Elderberry Lime kombucha. Ninja Golden Berry kombucha, Blue Ridge Bucha Ginger Hibiscus 1, and Blue Ridge Bucha Ginger Hibiscus 2 did not overlap with any other sample groups, indicating that each product contained distinct VOCs not present in the other products leading to class separation on both principal components plotted. Ninja Cranberry Lime Ginger kombucha, confidence ellipse overlaps with Synergy kombucha 2. Sage Mermaid Ginger Lemon kombucha, Synergy kombucha 1, Ninja Cranberry Lime Ginger kombucha, and Synergy kombucha 2 all group closely, indicating a common set of VOCs. These four kombucha products contained ginger as an additive ingredient for flavoring listed on the packaging, and the analyte zingiberene, a naturally occurring compound in ginger, was detected in all four. Data from the loadings plot (Figure S1) demonstrates a pattern in the type and complexity of molecules driving dispersion on the PCA scores plot. Locally or homemade-produced kombucha tended to contain simple alcohols and acids while more commercial brands like synergy contained terpenes and aromatic compounds. The terpenes and aromatic compounds present in the commercial brands could also come from the addition of fruit juices to the final kombucha products [34].

A similar PCA analysis was conducted on the one-dimensional data (Figure S2). All of the sample groups in the 1D PCA analysis were in the same position relative to one another as in the 2D PCA analysis. The corresponding loadings plot demonstrated a similar pattern to that found in the 2D analysis with more commercial brands containing terpenes and aromatics while the more local brands contained simpler acids and alcohols. Despite similar results, the 1D PCA scores plot had less variance along component 1 and more variance in component 2 than in the 2D PCA scores plot. The discrepancy between the two analyses probably resulted from the different volatile profiles contributing to the 1D and 2D PCA scores plots.

**Fig. 6:**
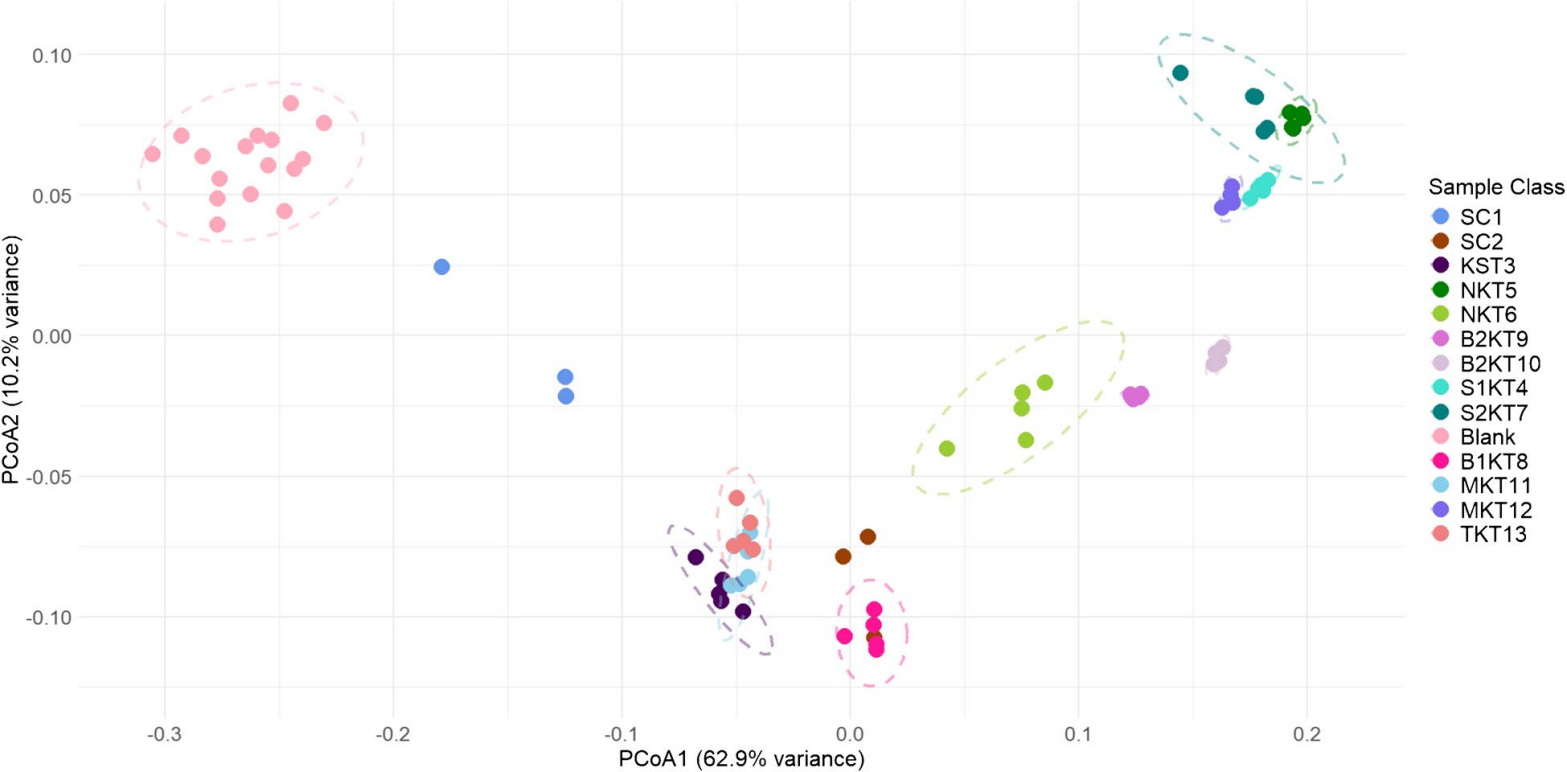
Principal coordinate analysis (PCoA) scores plot with Bray-Curtis dissimilarity, logarithmic transform, and 95% confidence ellipses for collected comprehensive two-dimensional gas chromatographic data. Sample groups shown include SCOBY 1 (SC1), SCOBY 2 (SC2), Blue Ridge Bucha Starter Tea (KST3), Synergy 1 (S1KT4), Ninja Kombucha Cranberry Ginger Lime (NKT5), Ninja Kombucha Goldenberry (NKT6), Synergy 2 (S2KT7), Blue Ridge Bucha Elderberry Lime (B1KT8), Blue Ridge Bucha Ginger Hibiscus 1 (B2KT9), Blue Ridge Bucha Ginger Hibiscus 2 (B2KT10), Sage Mermaid Blueberry Pomegranate (MKT11), Sage Mermaid Ginger Lemon (MKT12), Family tea (TKT13), and Blanks

While PCA is a common technique for dimensionality reduction, especially in GC×GC-TOFMS data, it is limited to measuring similarities and differences in the data using Euclidean distances. Euclidean distances are not necessarily ideal for measuring variance in biological samples due to the absence of upper limits and the fact that it is double zero symmetric (i.e., adding features with zero values is equivalent to adding features of any constant value, so zeros are unbiased by the analysis), both of which could skew the data to show more variance than is true in reality [33]. Another common ordination technique used on biological data is principal coordinate analysis (PCoA). PCoA operates similarly to PCA in that it uses observed variables to create synthetic variables which can be plotted to represent similarity and dissimilarity of the samples. PCoA differs from PCA in that it is not confined to Euclidean distances but can use non-Euclidean distance matrices such as Bray-Curtis dissimilarity [33]. Non-Euclidean similarity is a helpful tool for analyzing volatilomics data, as it emphasizes relative differences in compound presence and abundance rather than absolute intensity, making it better suited for comparing complex VOC profiles. Bray-Curtis dissimilarity ranges from 0 to 1 where 0 represents no overlap between sample groups and 1 represents complete overlap between sample groups. The resulting range of values is fed into the PCoA matrix to generate a PCoA scores plot similar to a PCA scores plot (Fig. 6).

The generated PCoA scores plot was very similar to the previously discussed PCA scores plot. All of the sample groups were in the same position relative to one another as in the PCA scores plot. Intrasample variation appears lower in the PCoA scores plot compared to the PCA scores plot, resulting in narrower confidence ellipses. Overall, the PCoA scores plot demonstrated the same relationships between sample groups as the PCA scores plot, possibly due to the log transformation placed on the data before PCA and PCoA analysis. The loadings for the two-dimensional PCoA analysis are shown in Figure S3, and it demonstrates a similar pattern to that of the PCA scores plot. The similarities seen between the two techniques demonstrate the effectiveness of using PCoA for dimensionality reduction of GC×GC data.

**Fig. 7:**
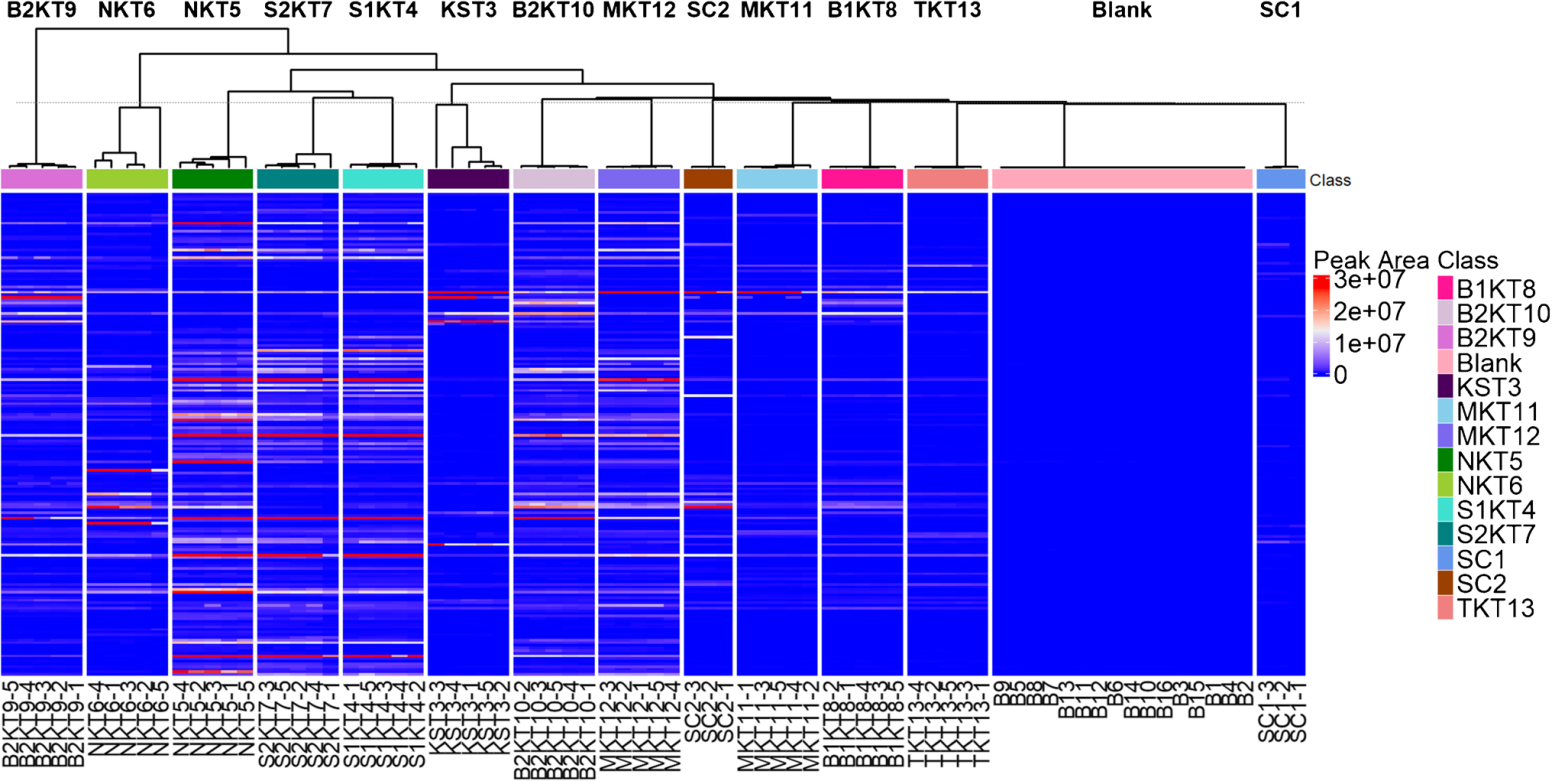
Heatmap based on peak areas combined with hierarchical cluster analysis (HCA) using data collected via GC×GC-TOFMS. Sample groups shown include SCOBY 1 (SC1), SCOBY 2 (SC2), Blue Ridge Bucha Starter Tea (KST3), Synergy 1 (S1KT4), Ninja Kombucha Cranberry Ginger Lime (NKT5), Ninja Kombucha Goldenberry (NKT6), Synergy 2 (S2KT7), Blue Ridge Bucha Elderberry Lime (B1KT8), Blue Ridge Bucha Ginger Hibiscus 1 (B2KT9), Blue Ridge Bucha Ginger Hibiscus 2 (B2KT10), Sage Mermaid Blueberry Pomegranate (MKT11), Sage Mermaid Ginger Lemon (MKT12), Family tea (TKT13), and Blanks

Another common tool for analyzing complex data matrices is hierarchical cluster analysis (HCA). HCA is an unsupervised technique that groups samples based on their nearness or similarity without any dimensionality reduction [35]. It produces a grid-like heatmap plot shown with a dendrogram to describe class similarities (Fig. 7).

There was tight clustering observed between Ninja Goldenberry kombucha, Ninja Cranberry Lime Ginger kombucha, Synergy kombucha 1, and Synergy kombucha 2. Tight clustering suggests that all four kombucha samples contain many of the same VOCs in similar amounts as well as lack the same VOCs in similar amounts. There was a similar relationship observed between the four products in the PCA and PCoA analyses, exempting Ninja Goldenberry kombucha which grouped on its own. Weaker clustering between Blue Ridge Bucha Ginger Hibiscus 1, the starter tea, and Blue Ridge Bucha Ginger Hibiscus 2 indicated the presence and absence of similar compounds, but with different abundances. Sage Mermaid Blueberry Pomegranate kombucha, Blue Ridge Bucha Elderberry Lime kombucha, Family kombucha, and SCOBY 1 are not observed to be in any tight clusters, meaning they contain more unique volatile components compared to other samples. Groupings differ between HCA, PCA, and PCoA due to the difference in dimensionality reduction. PCoA reduces dimensionality to plot multivariate data on a 2D surface which could change how sample groups are visualized. HCA uses the full dissimilarity matrix to make stepwise, local comparisons and builds a dendrogram based on relative similarity, without reducing the data’s dimensionality. Samples that overlap in PCoA can still be clustered separately in HCA, depending on how similar they are across all measured dimensions. A similar HCA heatmap analysis was performed on the 1D GC data (Figure S4) which resulted in less clustering overall, likely due to the fewer number of volatiles included in the analysis.

For the discrimination of each different class of kombucha product, the Electronic Supplementary Material provides peak identity information for each component (Table S1) and information about which component was identified across each kombucha class (Table S2). Additional loadings plots from each analysis performed are also included in the Electronic Supplementary Material.

### Differentiating tea volatiles and microbial volatiles

In order to differentiate between VOCs generated by the tea matrix and VOCs generated by the microbial matrix, fold change statistical tests were performed. The goal of a fold change test is to establish a large relative change between two samples or groups of samples, essentially establishing the key volatiles that differentiate one sample group from another. Fold change was applied in two ways to investigate what VOCs distinguished microbial samples from kombucha samples. The inclusion of flavorings to the kombucha during the production process complicates the allocation of VOCs to tea or microbial processes. Assigning any VOC as a microbial product or tea product in the following discussion adhered to prevalent literature as references in the text. It could also be possible to conclude that any VOC associated with flavoring would cluster with the tea volatiles on the basis of how the fold change test operates.

**Fig. 8:**
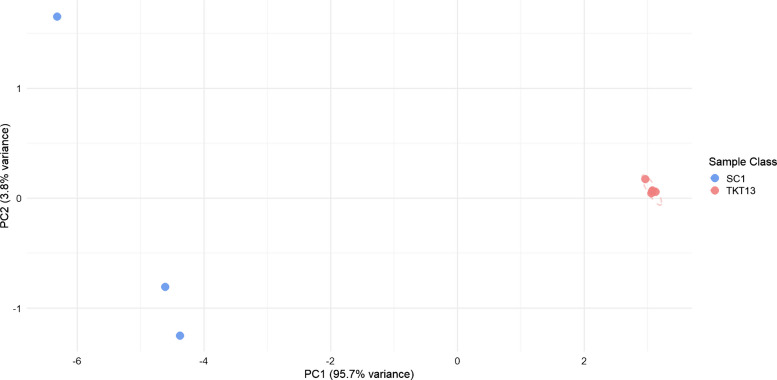
Principal component analysis (PCA) scores plot with logarithmic transform and mean centering for visualization of the fold change test performed on SCOBY 1 (SC1) and Family kombucha (TKT13)

A fold change test was performed to compare SCOBY 1 to the Family kombucha, the product produced by SCOBY 1. A PCA scores and loadings plot was generated to visualize the results of the fold change test (Fig. 8).

Clear separation was observed between the SCOBY 1 sample group and the Family kombucha sample group on the PCA scores plot, driven by several key volatile compounds. From the PCA loadings plot (Figure S5), 2-methylbutanoic acid, 3-methylbutanoic acid, and ethyl 2-methylpropanoate were found at a much higher abundance in the SCOBY 1 sample group than the family kombucha sample group. 2-methylbutanoic acid, 3-methylbutanoic acid, and ethyl 2-methylpropanoate are all known metabolites of SCOBY microorganisms described in literature and as found in the microbial VOC (mVOC) database, a database that contains extensive literature on microbial volatile organic compounds [20, 36]. 2-Methylbutanoic acid, 3-methylbutanoic acid, and ethyl 2-methylpropanoate can be produced by *Saccharomyces cerevisiae*, a common brewing yeast. 3-methylbutanoic acid and ethyl 2-methylpropanoate are also recorded as metabolites of *Pichia fermentans*, another known SCOBY constituent. Additionally, ethyl 2-methyl propanoate is a metabolite of *Brettanomyces bruxellensis*, a typical yeast found in most kombucha studies including a culture-based study of German kombucha samples [2, 20].

VOCs that were found in high abundance in the Family kombucha samples compared to the SCOBY 1 sample group include butyl acetate, hexanoic acid, *p*-xylene, hexan-1-ol, 3-methylbutyl acetate, dodecan-1-ol, α-terpineol, ethyl octanoate, 3-methylbutan-1-ol, decanoic acid, nonanoic acid, ethyl decanoate, linalool, 2-phenylethanol, octanoic acid, and 2-ethylhexan-1-ol. While terpenes and terpenoids are generally thought of as plant-associated compounds, recent studies have shown that some bacteria and yeast contain terpene synthase (TPS), an enzyme that catalyzes the cyclization of oligomers to terpenes [37]. The terpenes identified through the fold change statistical test comparing SCOBY 1 to Family tea were more abundant in the Family tea, so it is reasonable to conclude that α-terpineol and linalool are plant products rather than microbial metabolites in this case. The acids, esters, and alcohols found at a higher abundance in the tea sample are all possible microbial metabolites [36]. VOCs do not tend to associate with a single source; therefore, it is difficult to attribute any of the more common volatiles with a specific sample type. Furthermore, some of the VOCs present could be due to additional flavoring after the fermentation process during kombucha production.

**Fig. 9:**
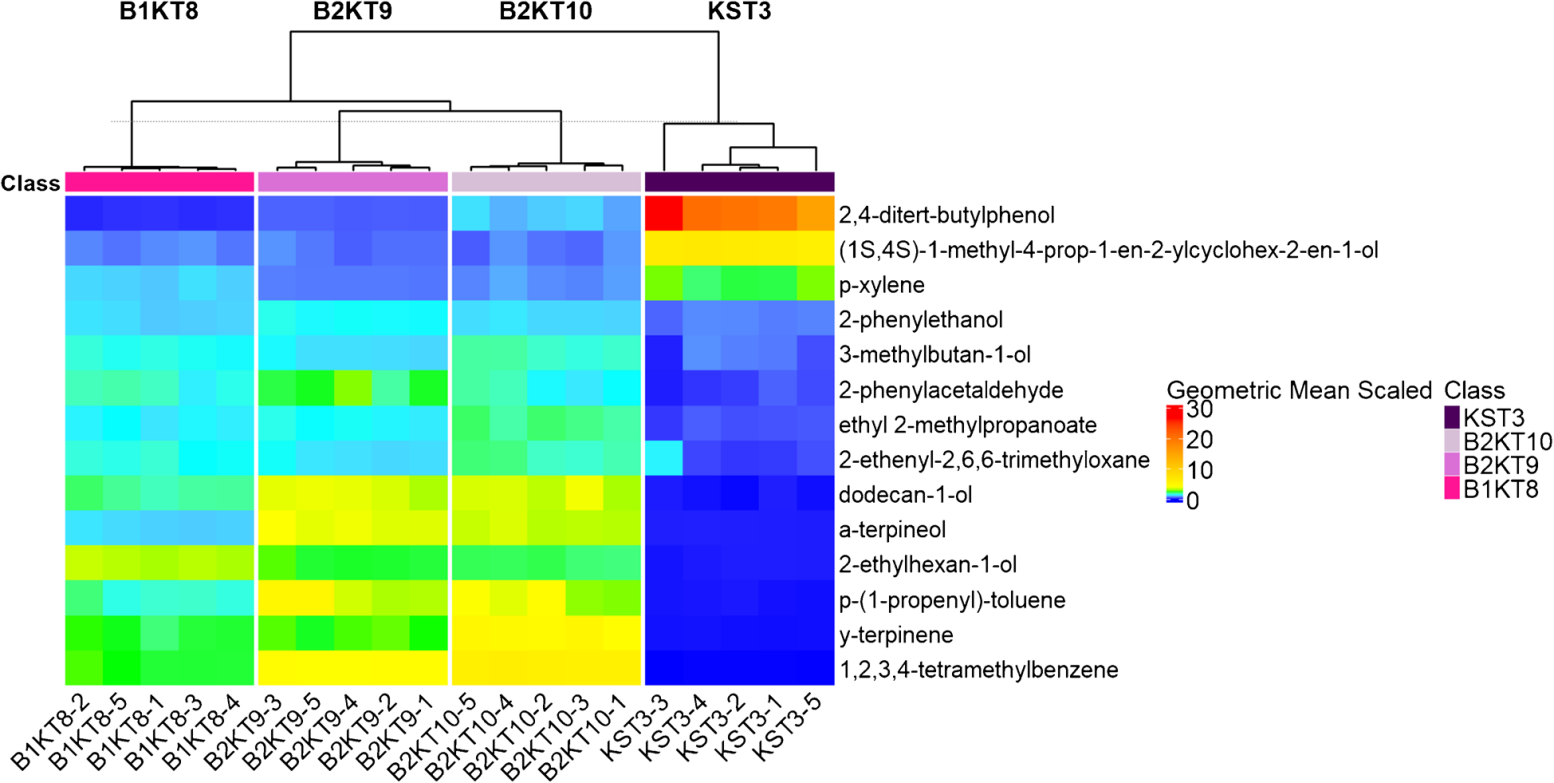
Heatmap representation with geometric mean scaling applied to fold change statistical test performed on Blue Ridge Bucha starter tea vs Blue Ridge Bucha kombucha tea products. Sample groups shown include Blue Ridge Bucha Starter Tea (KST3), Blue Ridge Bucha Elderberry Lime (B1KT8), Blue Ridge Bucha Ginger Hibiscus 1 (B2KT9), and Blue Ridge Bucha Ginger Hibiscus 2 (B2KT10)

Another fold change statistical test was performed on the Blue Ridge Bucha kombucha products. All of the Blue Ridge Bucha kombucha products were categorized as a single group for the purposes of the fold change test and were compared to the Blue Ridge Bucha starter tea. The Blue Ridge Bucha starter tea was pulled from a batch of kombucha that had not finished fermenting, and no flavorings were added, so it was treated as a SCOBY sample for the fold change analysis. A heatmap was generated to visualize the results of the fold change test between the Blue Ridge Bucha starter tea and the Blue Ridge Bucha kombucha products (Fig. 9).

The fold change test showed that Blue Ridge Bucha starter tea differed from each of the Blue Ridge Bucha kombucha products. The VOCs 2,4-ditert-butylphenol, 1-methyl-4-prop-1-en-2-ylcyclohex-2-en-1-ol, and *p*-xylene were more abundant in the Blue Ridge Bucha starter tea than in the kombucha products. 2,4-ditert-butylphenol is listed as a *S. cerevisiae* metabolite on the mVOC database [36]. There is also an entry for *p*-xylene in the mVOC database where it is listed as a metabolite of *Pichia kluyveri*, a film-forming yeast used in wine and beer as well as studied in kombucha [21]. 1-methyl-4-prop-1-en-2-ylcyclohex-2-en-1-ol is not listed in the mVOC database, but it is a recorded volatile from brewer’s spent grains, leftover grain material from the beer brewing process, analyzed by pyrolysis gas chromatography mass spectrometry (Py-GC-MS) [38].

VOCs that were found to be more abundant in the Blue Ridge Bucha kombucha tea group were 2-phenylethanol, 3-methylbutan-1-ol, 2-phenylethylacetaldehyde, ethyl 2-methyl propanoate, 2-ethenyl-2,6,6-trimethyloxane, dodecan-1-ol, α-terpineol, 2-ethylhexan-1-ol, *p*-(1-propenyl)-toluene, γ-terpinene, and 1,2,3,4-tetramethylbenzene. Similar to the SCOBY 1 and Family kombucha fold change analysis, all the volatiles detected at higher abundances in the tea sample could also be produced by microorganisms except 2-ethenyl-2,6,6-trimethyloxane and *p*-(1-propenyl)-toluene [36]. 2-ethenyl-2,6,6-trimethyloxane was detected in black tea in a 1984 study using high-vacuum distillation and adsorption chromatography, and so it is a reasonable VOC to find in kombucha [39]. *p*-(1-propenyl)-toluene was found to be a volatile component of passion fruit which suggests it might also be present in other foods and beverages [40]. Like the previous fold change results, it is possible that some of the volatiles associated with the kombucha tea products were present due to additional flavorings added during the production process.

Notably, ethyl 2-methylpropanoate was determined as a SCOBY volatile in the SCOBY 1 and Family kombucha fold change analysis, but it was determined as a tea volatile in the Blue Ridge Bucha fold change analysis. It is possible that ethyl 2-methylpropanoate is a volatile component of both SCOBY and kombucha tea. The identification of ethyl 2-methylpropanoate as a VOC in both the SCOBY and the tea emphasizes the limitations of relying solely on volatile profiles to determine a product’s origin and highlights the necessity to further develop GC×GC-TOFMS analyses for the comprehensive characterization of sample-specific volatile fingerprints.

Data within the study were collected using software which was designed for its analysis, using the suite of LECO ChromaTOF software products. It is common in chromatography to use a data processing platform that is provided with the instrument, since software is designed to handle artifacts specifically from that brand of instrumentation. However, it should be noted that all peak table data from each software can additionally be exported and assessed in other platforms, such as R Statistical Software, MATLAB, or Python. Additionally, there are other software platforms which can accept.cdf files, as well as increasing open-source coding options to evaluate GC×GC-TOFMS data. It is possible that assessing the software on another platform may lead to differences in features identified.

## Conclusion

The goal of this study was threefold in comparing the efficacy of GC-TOFMS to GC×GC-TOFMS, using VOC data to differentiate between kombucha products, and establishing an understanding of the VOCs composing the kombucha tea VOC contribution versus the kombucha microbial VOC contribution. While both GC-TOFMS and GC×GC-TOFMS were successful in separating analytes, GC×GC-TOFMS was able to resolve complex coelutions that GC-TOFMS could not. GC-TOFMS detected 127 analytes across 15 compound classes while GC×GC-TOFMS detected 182 analytes across 17 compound classes. Data analysis techniques like PCA, PCoA, and HCA were successful in determining similarities and differences between kombucha products based on logarithmically scaled peak areas. Locally or homemade-produced kombucha products tended to contain simple alcohols and acids while more commercial brands like Synergy contained terpenes and aromatic compounds. The use of fold change to examine the kombucha microbial matrix and the kombucha tea matrix emphasized the need for method development using GC×GC-TOFMS to detect more characteristic volatile compounds. Future studies could explore microbial composition through microbial genetic sequencing. Data from genetic sequencing in combination with VOC data could expand our understanding of the nature of kombucha products.

This study is significant in its comparison of traditional analytical methods with novel developments. In understanding the values and limitations of both chromatographic systems, analytical chemists can make informed decisions about which technique suits the aim of their experimentation. Moreover, understanding the composition of increasingly popular products such as kombucha can contribute to the knowledge around its nutritional impact on consumers and could potentially play a role in future regulatory efforts for kombucha production.

## Supplementary Information

Below is the link to the electronic supplementary material.

Supplementary Material 1 (PDF 1.26 MB)

## Data Availability

The data are available from the corresponding author upon reasonable request.
