# Supplementary material for: Evaluating chromatographic techniques for the aroma profiling of kombucha
Source: Anal Bioanal Chem. 2025 Dec 15;418(10):3059–73. doi: 10.1007/s00216-025-06257-5 (PMC13144191; doi:10.1007/s00216-025-06257-5)
Supplement: Supplementary file 1 — Supplementary Material 1 (PDF 1.26 MB) [file 216_2025_6257_MOESM1_ESM.pdf]

Table S1. A comprehensive list of all the analytes detected using both GC-TOFMS (1D) and GC×GC-TOFMS (2D). For both techniques, the median retention time(s) (RT) for each analyte is listed, including both the primary and secondary RTs for the 2D analysis. Analytes that were not detected in the 1D analysis are denoted by a "-" in the Median 1D RT column. Both experimental retention index (RI) values and reference RI values from NIST spectral library are included for identification confirmation. The ± symbol represents the range of retention index values reported for each analyte in NIST. The asterisk (*) preceding any library RI value indicates that only estimated RI values for a semi-standard nonpolar column were reported for that analyte. Confirmation of analyte identity by standard injection is denoted by an asterisk (*) following the analyte's name.

	**Median 2D RT (s)**		**Median 1D RT (s)**	**Retention Index**	
**Analyte**	**RT1 (s)**	**RT2 (s)**	**RT1 (s)**	**Library**	**Experimental**
2-methylbutanal	358.0	1.71	366.93	662±8	666.7
pentan-2-one	394.0	1.88	392.98	685±7	689.7
ethyl propanoate	422.0	1.77	422.83	710±4	707.7
propyl acetate	426.0	1.82	426.81	708±8	712.8
3-methylbutan-1-ol	454.0	1.78	453.83	736±5	730.8
pent-3-en-2-one	466.0	2.12	463.35	733±6	728.2
ethyl 2-methylpropanoate	498.0	1.80	493.64	756±5	738.5
toluene	510.0	1.77	508.97	764±8	756.4
isobutyl acetate	518.0	1.86	517.68	771±7	764.1
ethyl butanoate	566.0	1.91	563.30	802±3	800.0
butyl acetate	590.0	1.95	587.08	812±4	814.6
ethyl 2-hydroxypropanoate	590.0	2.27	585.86	815±6	814.6
furfural	630.0	2.62	-	833±4	833.3
1-ethyl-5,5-dimethylcyclopenta-1,3-diene	646.0	1.73	-	856.00	843.8
3-methylbutanoic acid	654.0	1.82	641.03	858±18	845.8
ethyl 2-methylbutanoate	658.0	1.88	653.50	849±3	847.9
ethyl 3-methylbutanoate	662.0	1.88	658.97	854±3	850.0
hex-3-en-1-ol	670.0	2.09	665.64	857±3	854.2
2-methylbutanoic acid	670.0	1.80	688.21	860±15	854.2
ethylbenzene	682.0	1.97	-	855±10	860.4
hexan-1-ol	690.0	1.95	688.28	868±4	864.6

p-xylene	698.0	1.97	694.77	865±7	868.8
3-methylbutyl acetate	706.0	1.90	703.29	876±2	872.9
heptan-2-one	734.0	2.05	731.35	891±2	887.5
styrene	738.0	2.13	736.77	893±5	889.6
o-xylene	746.0	2.05	-	888±8	895.8
heptan-2-ol	750.0	1.93	747.91	900±4	897.9
ethyl pentanoate	754.0	1.96	748.82	900±2	900.0
2,7,7-trimethylbicyclo[2.2.1]hept-2-ene	770.0	1.74	-	906±7	908.0
1-(furan-2-yl)ethanone	778.0	2.68	-	911±4	914.0
unknown 04	782.0	2.14	779.47	-	924.0
1,7,7-trimethyltricyclo[2.2.1.02,6]heptane	806.0	1.76	-	925±2	926.0
cumene	810.0	2.00	-	922±9	930.0
α-pinene*	826.0	1.74	823.84	937±1	934.0
β-citronellene	842.0	1.68	839.00	943.00	944.0
camphene*	858.0	1.81	855.06	951±3	950.0
benzaldehyde	878.0	2.63	875.22	962±3	960.0
3-methylbutyl propanoate	886.0	1.97	-	969±3	964.0
2-ethenyl-2,6,6-trimethyloxane	894.0	1.91	896.34	972±5	970.0
hexanoic acid	902.0	1.96	905.58	990±16	972.0
phenol	906.0	2.37	903.98	981±5	976.0
6-methylhept-5-en-2-one	922.0	2.11	920.25	986±2	984.0
octan-2-one	930.0	2.06	-	991±3	986.0
6-methylhept-5-en-2-ol	934.0	2.04	-	993±4	990.0
β-myrcene*	934.0	1.80	929.91	991±2	990.0
dehydrocineole	938.0	1.95	935.32	991±2	992.0
ethyl hexanoate	946.0	1.93	942.03	999±3	996.0
octanal	954.0	2.02	-	1003±2	1000.0
α-phellandrene	966.1	1.88	961.73	1005±2	1006.4
3-methylbutyl 2-methylpropanoate	970.1	1.90	968.81	1014±4	1010.6
isocineole	986.1	1.91	982.48	1016±2	1014.6
unknown 08	990.1	1.88	985.47	-	1019.1

2-ethylhexan-1-ol	1002.1	1.90	1000.38	1030±3	1022.9
m-cymene	1006.1	1.97	1000.48	1022±8	1025.0
β-phellandrene	1014.1	1.88	1009.91	1031±2	1031.9
d-limonene*	1014.1	1.87	1009.43	1031±1	1031.9
eucalyptol	1022.1	1.97	1016.59	1032±2	1036.2
3,7-dimethylocta-1,3,6-triene*	1042.1	1.83	1040.50	1049±2	1046.8
2-phenylacetaldehyde	1042.1	2.68	1037.59	1045±4	1046.8
5-[but-2-en-2-yl]-2,2-dimethyloxolane	1042.1	1.90	-	*1018	1046.8
γ-terpinene*	1070.1	1.91	1066.78	1060±2	1058.3
unknown 06	1074.1	2.25	1070.30	-	1063.8
octan-1-ol	1082.1	1.94	1079.61	1070±3	1068.1
acetophenone	1086.1	2.69	1082.98	1066±4	1070.2
3-methylbenzaldehyde	1090.1	2.67	1085.65	1068±11	1072.3
linalool oxide	1098.1	2.02	1092.54	1074±4	1076.6
4-methylbenzaldehyde	1114.1	2.64	1112.43	1079±1	1085.1
nonan-2-one	1126.1	2.03	-	1092±2	1087.5
fenchone	1130.1	2.20	1127.92	*1121	1093.6
p-cymenene	1130.1	2.15	1125.49	1090±2	1093.6
ethyl heptanoate	1134.1	1.92	1130.71	1098±2	1095.7
methyl benzoate	1138.1	2.50	-	1094±3	1097.9
linalool*	1142.1	1.94	1138.40	1099±2	1100.0
nonanal	1150.1	2.00	1146.08	1104±2	1104.3
6-methylhepta-3,5-dien-2-one	1154.1	2.42	-	1107±1	1106.5
4-ethenyl-1,2-dimethylbenzene	1170.1	1.86	-	*1110	1115.2
2-phenylethanol	1174.1	2.61	1170.52	1116±5	1117.4
benzene derivative C10H12 06	1174.1	2.09	-	-	1117.4
fenchol	1178.1	2.07	1175.35	1114±4	1119.6
isoborneol methyl ether	1182.1	1.92	-	1117.00	1121.7
1-methyl-4-prop-1-en-2-ylcyclohex-2-en-1-ol	1194.1	2.29	-	1123±4	1124.4
2,6-dimethylocta-2,4,6-triene	1198.1	1.91	-	1131±2	1130.4
unknown 03	1198.1	2.16	1197.02	-	1130.4

1-terpineol	1214.1	2.10	-	1137±4	1139.1
2-phenylacetonitrile	1218.1	3.04	1217.32	1144±5	1141.3
benzene derivative C10H12 01	1222.1	2.08	-	-	1140.0
unknown 05	1222.1	1.65	-	-	1140.0
2-methylpropyl hexanoate	1234.1	1.90	-	1149±3	1150.0
β-terpineol	1238.1	2.10	1233.47	1153±9	1152.2
2-bornanone	1242.1	2.39	1237.54	1144±12	1154.3
1-ethenyl-4-methoxybenzene	1246.1	2.46	-	1156±2	1156.5
unknown, C10H16	1250.1	2.19	-	-	1158.7
4-ethylphenol	1262.1	2.40	1257.81	1169±3	1165.2
unknown 09	1262.1	2.19	1259.30	-	1165.2
2-methyl-acetophenone	1270.1	2.61	-	1173±4	1167.4
dimethylbenzeneethanol	1270.1	2.22	-	1158±3	1167.4
octanoic acid	1270.1	1.93	1266.88	1180±7	1167.4
borneol	1278.1	2.20	1274.71	1170±5	1171.1
ethyl benzoate	1278.1	2.41	-	1171±2	1171.1
1,2,3,4-tetramethylbenzene	1294.1	2.13	1292.82	1144±8	1182.6
p-(1-propenyl)-toluene	1306.1	2.38	1301.78	*1114	1189.1
ethyl octanoate	1314.1	1.88	1311.27	1196±3	1191.1
α-terpineol	1318.1	2.17	1314.92	*1143	1193.3
estragole	1334.1	2.35	1325.57	1196±1	1204.7
unknown 11	1338.1	1.88	1334.00	-	1207.0
unknown 13	1346.1	2.15	-	-	1209.5
4-ethenoxy-2,6,6-trimethylbicyclo[3.1.1]hept-2-ene	1354.1	1.88	-	*1174	1214.3
citronellol	1374.1	2.01	1368.48	1228±3	1227.9
unknown 12	1386.1	1.90	-	-	1233.3
ascaridole	1390.1	2.13	1385.59	1242±5	1237.2
benzene derivative C10H12 02	1394.1	2.05	1391.34	-	1239.5
2-methylbutyl hexanoate	1406.1	1.87	1409.47	1247±1	1245.2
ethyl 2-phenylacetate	1406.1	2.49	1402.95	1247±3	1245.2
carvone	1414.1	2.37	1409.32	1246±4	1250.0

2,7-dimethylocta-2,6-dien-1-ol	1418.1	2.09	-	*1228	1253.4
1,3-ditert-butylbenzene	1426.1	1.89	-	1249.00	1257.1
benzene derivative C10H12 03	1430.1	2.30	1424.64	-	1259.5
nonanoic acid	1430.1	1.96	1428.89	1273±7	1259.5
2-phenylethyl acetate	1434.1	2.47	1424.53	1258±2	1261.9
terpin	1442.1	2.00	1439.61	1279.00	1267.4
4-ethyl-2-methoxyphenol	1470.1	2.44	-	1282±4	1283.7
anethole	1482.1	2.47	-	1287±2	1290.7
ethyl nonanoate	1486.1	1.88	1481.63	1296±2	1292.9
isobornyl acetate	1486.1	2.09	-	1286±2	1292.9
undecan-2-one	1486.1	1.97	-	1294±2	1292.9
undecan-2-ol	1494.1	1.85	1491.53	1303±4	1297.6
benzene derivative C10H12 04	1498.1	2.03	-	-	1300.0
6,7-dimethyl-1,2,3,5,8,8a-hexahydronaphthalene	1514.1	2.12	1512.54	*1273	1310.0
x,x,x-trimethylbenzaldehyde	1514.1	2.57	-	-	1310.0
2-methylpropyl octanoate	1570.1	1.83	1566.87	1348±1	1345.0
unknown 07	1570.1	2.21	1563.99	-	1345.0
3-hydroxyacetophenone	1574.1	1.84	1569.21	1439.00	1347.5
neric acid	1578.1	2.23	-	1344±8	1350.0
8,9-dehydrocycloisolongifolene	1586.1	1.88	-	1317±4	1356.4
decanoic acid	1598.1	2.01	1590.22	1373±7	1362.5
2-methylpropyl 3-hydroxy-2,2,4-trimethylpentanoate	1626.1	2.07	-	*1331	1380.0
cyclosativene	1630.1	1.88	1626.39	1368±3	1384.6
ethyl dec-9-enoate	1634.1	1.94	1630.62	1388±1	1387.2
copaene	1642.1	1.88	1637.42	1376±2	1392.3
benzene derivative C10H12 05	1646.1	2.23	-	-	1394.9
damascenone	1646.1	2.26	1643.39	1386±5	1394.9
ethyl decanoate	1646.1	1.86	1642.82	1396±2	1394.9
2-phenylethyl 2-methylpropanoate	1658.1	2.31	-	1396±1	1400.0
elemene	1662.1	1.90	-	1338±2	1405.3
unknown 10	1698.1	1.90	-	-	1426.3

2,6,6,8-tetramethyltricyclo[5.3.1.01,5]undec-8-ene	1706.1	1.97	1704.19	1410±2	1431.6
isocaryophyllene	1714.1	1.97	-	1407±3	1436.8
2,5,8-trimethyl-1,4-dihydronaphthalene	1722.1	2.32	1718.09	*1465	1442.1
2-phenylethyl butanoate	1726.1	2.38	1725.75	1444±4	1444.7
β-bergamotene	1726.1	1.93	1724.04	*1425	1444.7
α-bergamotene	1730.1	1.87	-	1435±5	1450.0
1-methyl-4-(6-methylheptan-2-yl)benzene	1738.1	1.91	1735.54	1449.00	1455.3
β-farnesene	1750.1	1.83	1744.64	1457±2	1460.5
β-acoradiene	1758.1	1.97	1753.45	1473±2	1465.8
dodecan-1-ol	1766.1	1.90	1765.44	1474±3	1473.7
amorphadiene	1774.1	2.00	1768.77	1457±3	1476.3
γ-himachalene	1782.1	2.01	1776.74	1477±5	1481.6
α-curcumene	1794.1	2.01	1792.67	1483±3	1489.5
zingiberene	1814.1	1.92	1810.80	1495±2	1505.6
x,x-dimethylnaphthalene	1818.1	2.30	1814.87	-	1508.3
2,4-ditert-butylphenol	1830.1	2.14	1826.66	1514±5	1513.9
β-bisabolene	1830.1	2.04	1831.31	1509±3	1513.9
γ-cadinene	1830.1	2.05	1827.40	1513±2	1513.9
2,6-ditert-butyl-4-methylphenol	1842.1	2.12	1837.20	1513±5	1522.2
himachalene	1846.1	2.11	-	1542.00	1525.0
2,6,10,10-tetramethyl-11-oxatricyclo[7.2.1.01,6]dodecane	1854.1	2.13	-	1499±3	1530.6
sesquiphellandrene	1858.1	1.94	1855.60	1524±2	1536.1
calamenene	1866.1	2.08	1862.76	1523±5	1541.7
1,4,4,7-tetramethyl-2,3,3a,5a,8,9-hexahydro-1H-cyclopenta[c][1]benzofuran	1874.1	2.18	1871.74	1540±4	1544.4
β-vatirenene	1898.1	2.20	1897.50	1541±13	1563.9
nerolidol	1910.1	1.98	1905.17	1543±14	1569.4
8-Isopropyl-1-methyl-3-methylenetricyclo[4.4.0.02,7]decan-4-ol	1934.1	2.21	-	1586±5	1586.1
ethyl dodecanoate	1942.1	1.83	1939.66	1595±2	1594.4
unknown, C15H24	1990.1	2.21	1985.38	-	1626.5
β-acorenol	2014.1	2.22	2013.09	1636±1	1647.1
2-phenylethyl hexanoate	2022.1	2.29	2020.28	1650±5	1652.9

α-eudesmol	2026.1	2.22	2022.61	1653±2	1655.9
cadalene	2038.1	2.26	-	1674±3	1661.8
cadinol	2054.1	2.27	2052.70	1640±2	1676.5
unknown 02	2074.1	2.40	-	-	1688.2
unknown 01	2118.1	2.36	-	-	1721.9
2-phenylethyl octanoate	2290.2	2.24	2287.88	1847±7	1858.1

Table S2. List of analytes detected using GC×GC-TOFMS according to their presence in each sample group as denoted by the "x". Sample groups include SCOBY 1 (SC1), SCOBY 2 (SC2), Blue Ridge Bucha Starter Tea (KST3), Synergy 1 (S1KT4), Ninja Kombucha Cranberry Ginger Lime (NKT5), Ninja Kombucha Goldenberry (NKT6), Synergy 2 (S2KT7), Blue Ridge Bucha Elderberry Lime (B1KT8), Blue Ridge Bucha Ginger Hibiscus 1 (B2KT9), Blue Ridge Bucha Ginger Hibiscus 2 (B2KT10), Sage Mermaid Blueberry Pomegranate (MKT11), Sage Mermaid Ginger Lemon (MKT12), and Family tea (TKT13).

Name	SC1	SC2	KST3	S1KT4	NKT5	NKT6	S2KT7	B1KT8	B2KT9	B2KT10	MKT11	MKT12	TKT13
1-methyl-4-prop-1-en-2-ylcyclohex-2-en-1-ol		x	x	x	x	x	x						
2,6,10,10-tetramethyl-11-oxatricyclo[7.2.1.01,6]dodecane				x	x		x					x	
3,7-dimethylocta-1,3,6-triene				x	x	x	x		x	x		x	
6-methylhepta-3,5-dien-2-one						x						x	
2,6-dimethylocta-2,4,6-triene				x	x	x	x		x	x		x	
β-farnesene				x	x		x		x	x		x	
pent-3-en-2-one			x	x			x			x			
calamenene				x	x		x		x	x		x	
hex-3-en-1-ol		x			x	x	x				x		
linalool oxide	x	x	x	x	x	x	x	x	x	x	x	x	x
1-(furan-2-yl)ethanone						x							
1,2,3,4-tetramethylbenzene				x	x	x	x	x	x	x		x	
1,3-ditert-butylbenzene					x	x							
1,4,4,7-tetramethyl-2,3,3a,5a,8,9-hexahydro-1H-cyclopenta[c][1]benzofuran				x	x		x					x	
1,7,7-trimethyltricyclo[2.2.1.02,6]heptane				x	x		x			x		x	
1-ethenyl-4-methoxybenzene				x		x							

1-ethyl-5,5-dimethylcyclopenta-1,3-diene				x	x	x	x		x	x		x	
1-methyl-4-(6-methylheptan-2-yl)benzene				x	x		x		x	x		x	
1-terpineol					x							x	
2,4-ditert-butylphenol	x	x	x	x			x					x	
2,5,8-trimethyl-1,4-dihydronaphthalene	x	x	x	x	x	x	x		x			x	x
2,6,6,8-tetramethyltricyclo[5.3.1.01,5]undec-8-ene				x	x		x		x	x		x	
2,6-ditert-butyl-4-methylphenol				x			x						
2,7,7-trimethylbicyclo[2.2.1]hept-2-ene					x		x						
2,7-dimethylocta-2,6-dien-1-ol				x	x	x	x		x	x		x	
2-bornanone				x	x	x	x		x	x		x	
2-ethenyl-2,6,6-trimethyloxane	x				x								x
2-ethylhexan-1-ol		x			x	x		x	x	x	x	x	x
2-methyl-acetophenone						x							
2-methylbutanal		x	x	x				x	x	x	x	x	
2-methylbutanoic acid	x		x					x	x	x			
2-methylbutyl hexanoate								x	x	x			
2-methylpropyl 3-hydroxy-2,2,4-trimethylpentanoate	x	x	x	x	x	x	x	x	x	x	x	x	x
2-methylpropyl hexanoate						x		x	x	x			
2-methylpropyl octanoate		x			x	x		x	x	x		x	
2-phenylacetaldehyde		x	x	x				x	x	x	x	x	x
2-phenylacetonitrile		x				x							
2-phenylethanol		x	x	x	x	x	x	x	x	x	x	x	x
2-phenylethyl 2-methylpropanoate			x					x	x	x			
2-phenylethyl acetate			x						x				
2-phenylethyl butanoate								x	x	x			
2-phenylethyl hexanoate								x	x	x			
2-phenylethyl octanoate								x	x	x			
3-hydroxyacetophenone				x	x		x					x	
3-methylbenzaldehyde		x		x									
3-methylbutan-1-ol		x	x	x	x	x	x	x	x	x	x	x	x
3-methylbutanoic acid	x	x	x				x	x	x	x			
3-methylbutyl 2-methylpropanoate		x	x			x	x	x	x	x			
3-methylbutyl acetate		x	x						x	x			
3-methylbutyl propanoate			x					x	x	x			

4-ethenoxy-2,6,6-trimethylbicyclo[3.1.1]hept-2-ene					x					x		x	
4-ethenyl-1,2-dimethylbenzene				x	x		x		x	x			
4-ethyl-2-methoxyphenol		x	x	x	x	x	x		x	x		x	x
4-ethylphenol				x	x	x	x						
4-methylbenzaldehyde		x		x									
8,9-dehydrocycloisolongifolene				x	x		x			x		x	
5-[(E)-but-2-en-2-yl]-2,2-dimethyloxolane					x								
6,7-dimethyl-1,2,3,5,8,8a-hexahydronaphthalene				x	x		x					x	
6-methylhept-5-en-2-ol				x	x		x		x	x		x	
6-methylhept-5-en-2-one				x	x		x		x	x		x	
α-bergamotene				x	x		x					x	
acetophenone		x		x	x	x	x	x	x	x	x	x	x
α-curcumene				x	x		x		x	x		x	
α-eudesmol				x	x		x		x	x		x	
amorphadiene				x	x		x		x	x		x	
anethole						x							
α-phellandrene				x	x	x	x		x	x		x	
α-pinene				x	x	x	x		x	x		x	
himachalene				x	x		x					x	
ascaridole	x	x	x	x	x	x	x		x			x	x
α-terpineol		x		x	x	x	x	x	x	x	x	x	x
β-acoradiene				x	x		x			x		x	
β-acorenol				x	x		x		x	x		x	
β-bergamotene					x		x		x	x		x	
β-bisabolene				x	x		x		x	x		x	
β-citronellene				x	x		x			x		x	
benzaldehyde	x	x	x	x			x	x	x	x	x		x
benzene derivative C10H12 01				x	x	x	x		x	x		x	
benzene derivative C10H12 02				x	x	x	x	x	x	x		x	
benzene derivative C10H12 03				x	x	x	x		x	x		x	
benzene derivative C10H12 04				x	x	x	x		x	x		x	
benzene derivative C10H12 05				x	x		x		x	x		x	
benzene derivative C10H12 06				x	x	x	x		x	x		x	
β-myrcene		x		x	x	x	x		x	x		x	

borneol				x	x		x		x	x		x	
β-phellandrene				x	x	x	x		x	x		x	
β-terpineol				x	x		x					x	
butyl acetate	x	x	x	x	x	x	x	x	x	x	x	x	x
β-vatirenene				x	x		x		x	x		x	
cadalene				x	x		x		x	x		x	
cadinol				x	x		x		x	x		x	
camphene				x	x		x		x	x		x	
carvone					x	x							
citronellol				x	x		x		x	x		x	
copaene				x	x		x		x	x		x	
cumene	x												x
cyclosativene				x	x		x		x	x		x	
damascenone		x	x	x	x	x	x	x	x	x			
decanoic acid				x	x		x						
dehydrocineole				x	x		x						
dimethylbenzeneethanol				x	x		x		x	x		x	
d-limonene				x	x	x	x	x	x	x		x	
dodecan-1-ol								x	x	x	x	x	x
elemene				x	x		x			x		x	
estragole						x							
ethyl 2-hydroxypropanoate					x	x							
ethyl 2-methylbutanoate		x		x	x	x	x	x	x	x	x	x	
ethyl 2-methylpropanoate	x	x	x	x	x	x	x	x	x	x		x	x
ethyl 2-phenylacetate		x	x	x	x	x	x	x	x	x	x	x	
ethyl 3-methylbutanoate		x	x	x	x	x	x	x	x	x	x	x	
ethyl benzoate		x		x	x	x	x		x	x		x	
ethyl butanoate		x		x	x	x	x	x	x	x	x	x	x
ethyl dec-9-enoate		x				x		x	x	x		x	
ethyl decanoate		x		x	x	x		x	x	x		x	x
ethyl dodecanoate		x		x	x	x		x		x	x	x	x
ethyl heptanoate		x		x	x	x		x	x	x	x	x	
ethyl hexanoate		x			x	x		x	x	x	x	x	x
ethyl nonanoate		x		x	x	x		x	x	x	x	x	x

ethyl octanoate		x		x	x	x	x	x	x	x	x	x	x
ethyl pentanoate		x		x	x	x						x	
ethyl propanoate			x					x	x	x			
ethylbenzene	x	x	x	x	x	x	x	x	x	x	x	x	x
eucalyptol				x	x	x	x		x	x	x	x	
fenchol				x	x		x	x		x		x	
fenchone						x							
furfural	x			x									
heptan-2-ol				x	x	x	x		x	x	x	x	
heptan-2-one		x	x	x	x	x	x	x	x	x	x	x	
hexan-1-ol			x	x	x	x	x	x	x	x	x	x	x
hexanoic acid	x	x	x	x	x	x	x	x	x	x	x	x	x
isoborneol methyl ether				x	x		x						
isobornyl acetate	x			x	x	x	x		x	x		x	x
isobutyl acetate		x	x					x	x	x			
isocaryophyllene				x	x		x			x		x	
isocineole					x							x	
8-Isopropyl-1-methyl-3-methylenetricyclo[4.4.0.02,7]decan-4-ol				x	x		x			x		x	
linalool		x	x	x	x	x	x	x	x	x	x	x	x
m-cymene				x	x	x	x			x		x	
methyl benzoate		x				x							
neric acid				x	x	x	x		x	x			
nerolidol				x	x		x			x		x	
nonan-2-one	x	x		x	x	x	x		x	x	x	x	
nonanal		x	x	x	x	x	x	x	x	x	x	x	x
nonanoic acid		x	x	x	x	x		x	x	x	x	x	x
octan-1-ol		x		x	x	x	x	x	x	x	x	x	x
octan-2-one			x	x	x	x	x		x	x	x	x	
octanal		x		x									
octanoic acid		x	x	x	x	x	x	x	x	x	x	x	x
o-xylene		x	x	x	x	x	x	x	x	x	x	x	x
p-(1-propenyl)-toluene				x	x	x	x	x	x	x		x	
p-cymenene				x	x	x	x	x	x	x		x	

pentan-2-one				x		x	x	x		x	x	x	
phenol								x	x	x	x	x	x
propyl acetate			x					x	x	x			
p-xylene	x	x	x	x	x	x	x	x	x	x	x	x	x
sesquiphellandrene				x	x		x		x	x		x	
styrene		x	x	x	x	x	x	x	x	x	x	x	x
terpin				x	x		x		x	x		x	
toluene				x	x		x						
undecan-2-ol				x	x		x		x	x		x	
undecan-2-one				x	x		x			x		x	
unknown 01				x	x		x		x	x		x	
unknown 02				x	x		x					x	
unknown 03				x	x		x			x		x	
unknown 04					x	x	x						
unknown 05				x	x	x	x		x			x	
unknown 06		x	x	x	x	x	x					x	
unknown 07				x	x		x						
unknown 08				x	x		x		x	x		x	
unknown 09				x	x		x		x	x		x	
unknown 10					x								
unknown 11				x	x		x			x		x	
unknown 12					x					x		x	
unknown 13				x	x		x			x		x	
unknown, C10H16				x	x		x		x	x		x	
unknown, C15H24				x	x		x			x		x	
x,x,x-trimethylbenzaldehyde		x		x				x	x	x	x	x	x
x,x-dimethylnaphthalene				x	x		x			x		x	
γ-cadinene				x	x		x		x	x		x	
γ-himachalene				x	x		x		x	x		x	
γ-terpinene				x	x		x			x		x	
zingiberene				x	x		x		x	x		x	

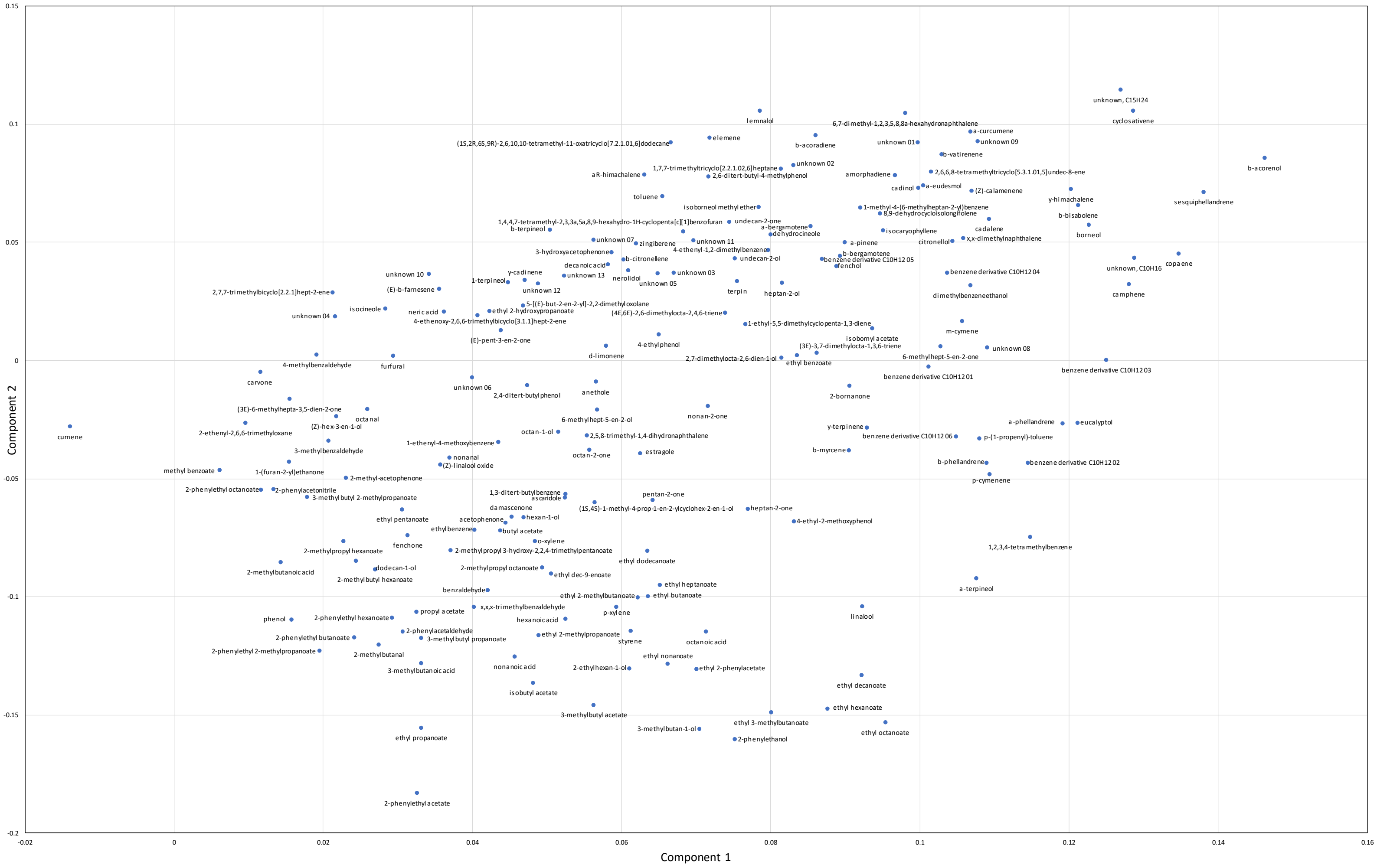

Figure S1. Principal Component Analysis (PCA) Loadings plot showing component 1 vs. component 2. Loadings correspond to how the data is dispersed in the PCA Scores plot.

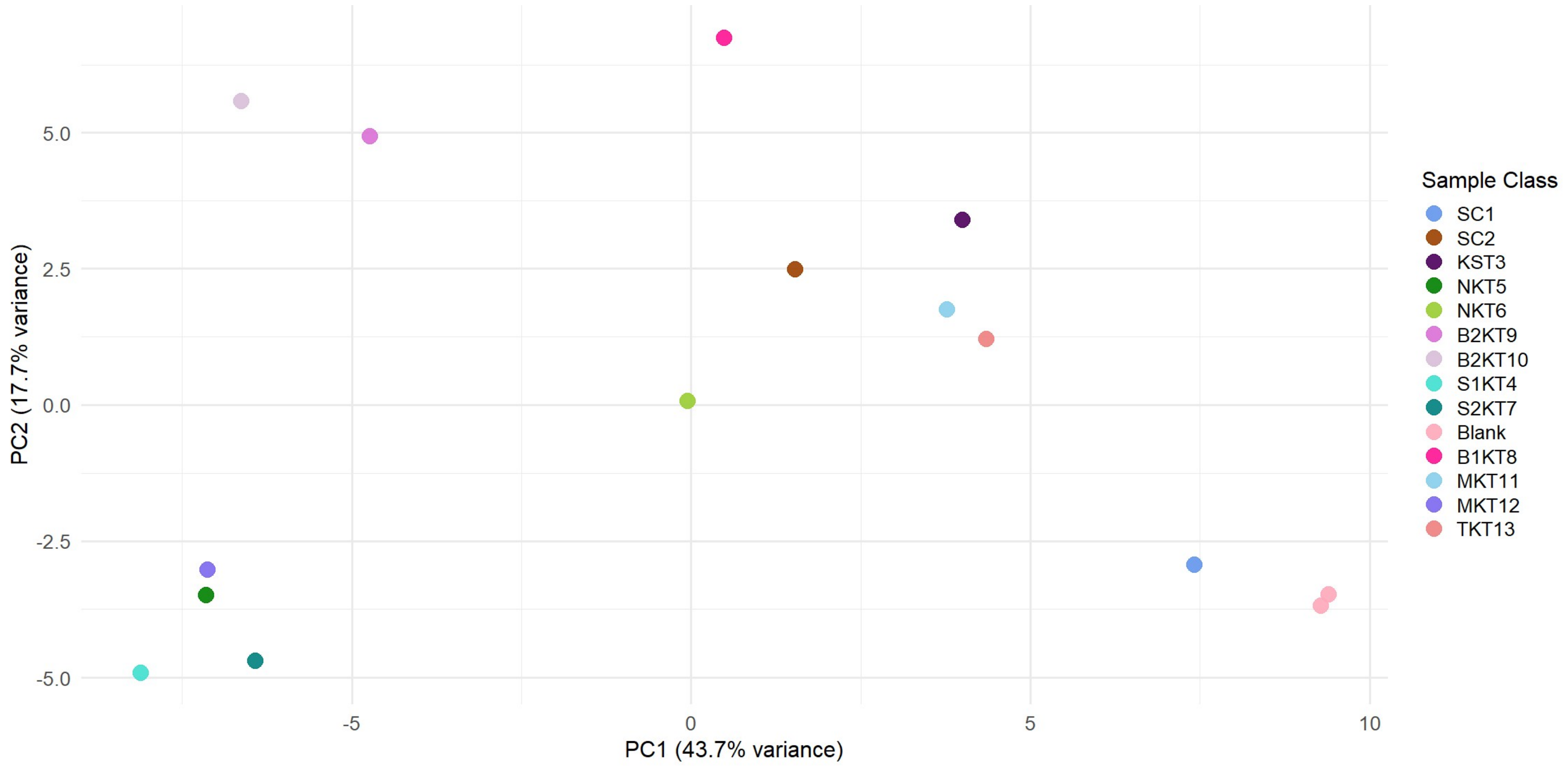

Figure S2. Principal Component Analysis (PCA) Scores plot with logarithmic transform, and mean-centering for collected 1D chromatographic data. Sample groups include SCOBY 1 (SC1), SCOBY 2 (SC2), Blue Ridge Bucha Starter Tea (KST3), Synergy 1 (S1KT4), Ninja Kombucha Cranberry Ginger Lime (NKT5), Ninja Kombucha Goldenberry (NKT6), Synergy 2 (S2KT7), Blue Ridge Bucha Elderberry Lime (B1KT8), Blue Ridge Bucha Ginger Hibiscus 1 (B2KT9), Blue Ridge Bucha Ginger Hibiscus 2 (B2KT10), Sage Mermaid Blueberry Pomegranate (MKT11), Sage Mermaid Ginger Lemon (MKT12), Family tea (TKT13), and blanks.

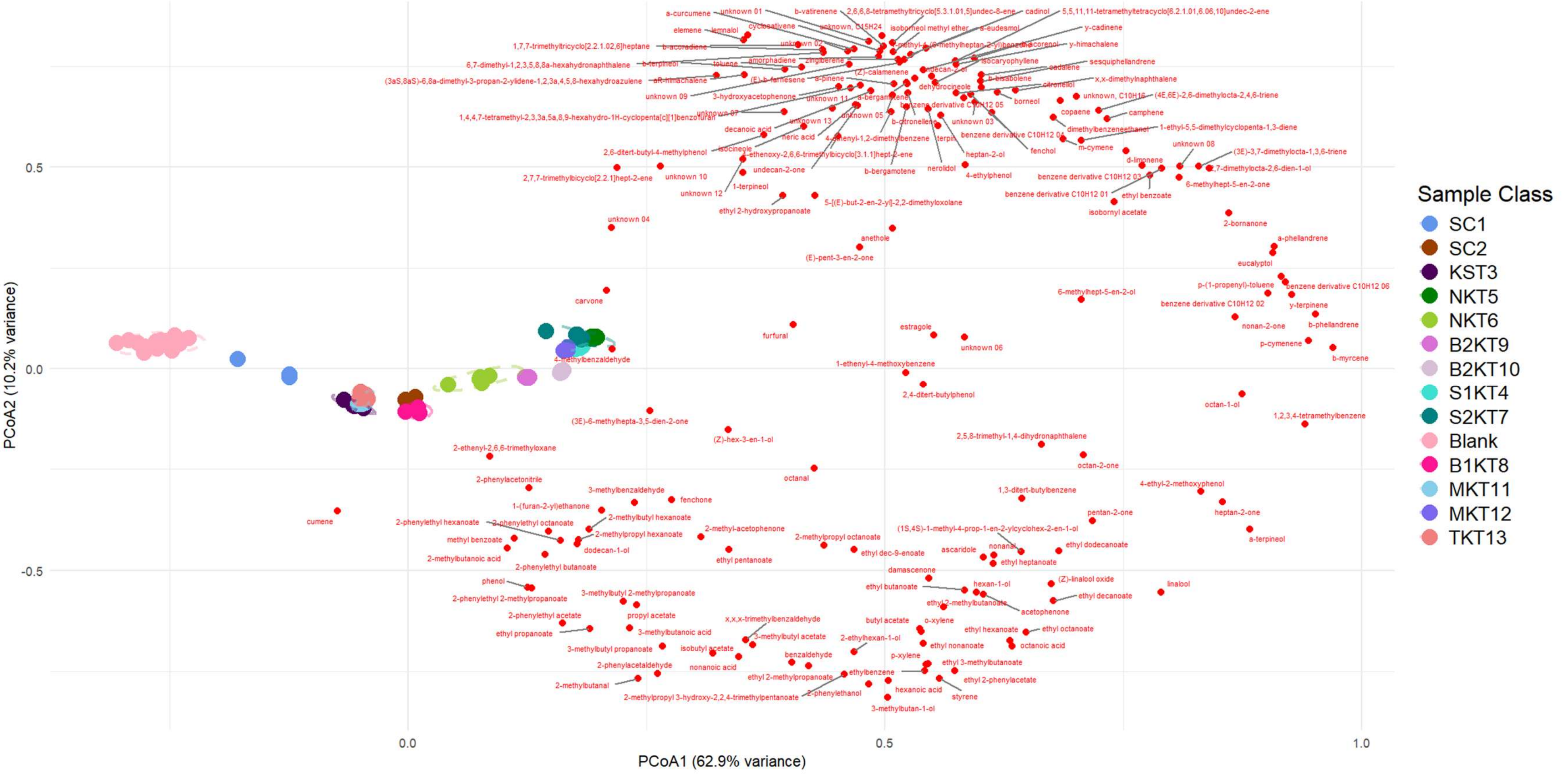

Figure S3. PCoA biplot including scores and loadings from the two-dimensional GC×GC-TOFMS PCoA analysis. Each data point represents a compound driving separation of sample groups along the coordinate axis. Sample groups include SCOBY 1 (SC1), SCOBY 2 (SC2), Blue Ridge Bucha Starter Tea (KST3), Synergy 1 (S1KT4), Ninja Kombucha Cranberry Ginger Lime (NKT5), Ninja Kombucha Goldenberry (NKT6), Synergy 2 (S2KT7), Blue Ridge Bucha Elderberry Lime (B1KT8), Blue Ridge Bucha Ginger Hibiscus 1 (B2KT9), Blue Ridge Bucha Ginger Hibiscus 2 (B2KT10), Sage Mermaid Blueberry Pomegranate (MKT11), Sage Mermaid Ginger Lemon (MKT12), Family tea (TKT13), and blanks.

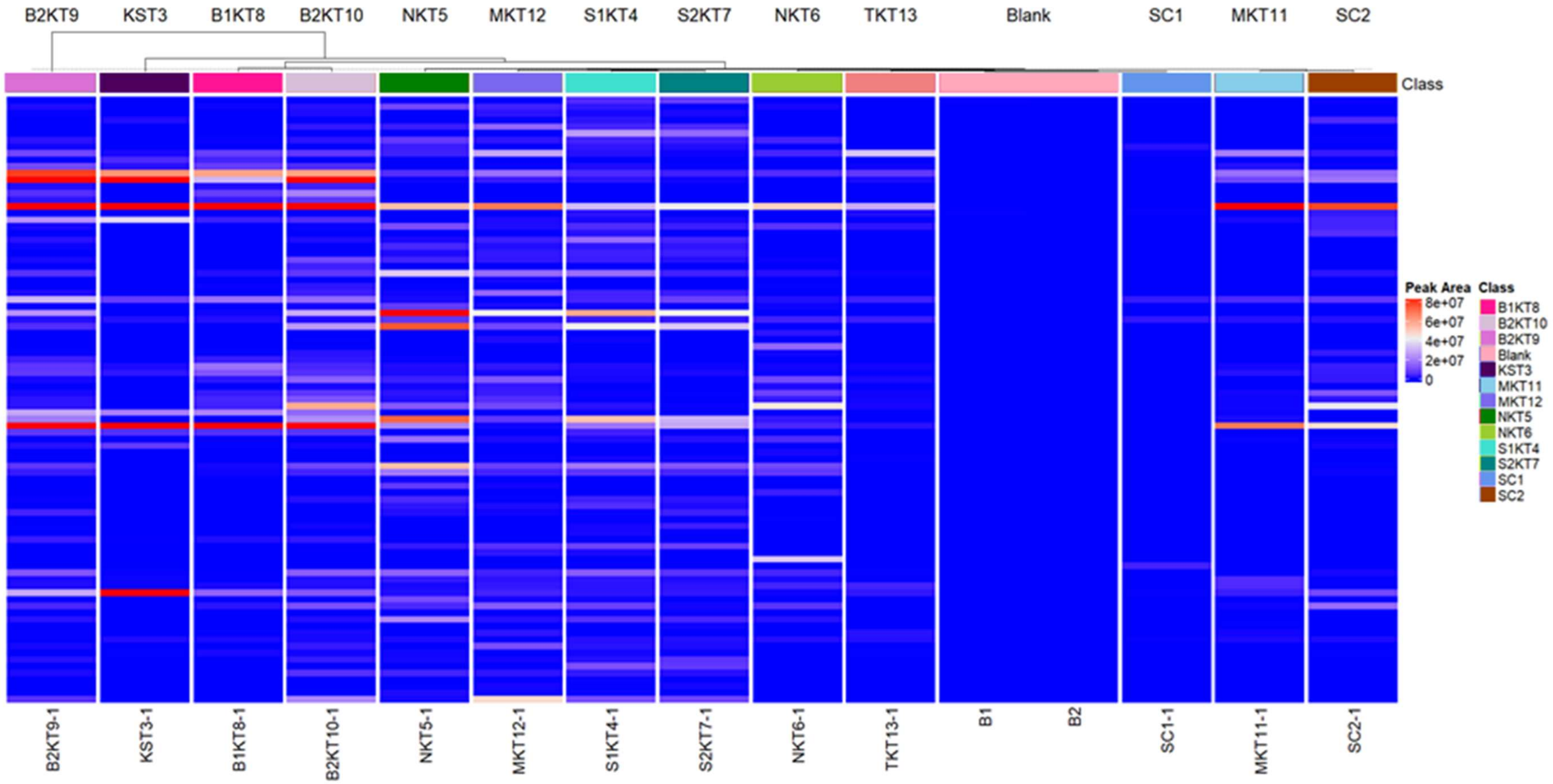

Figure S4. Heatmap based on peak areas combined with Hierarchical Cluster Analysis (HCA) using data collected via GC-TOFMS. Sample groups include SCOBY 1 (SC1), SCOBY 2 (SC2), Blue Ridge Bucha Starter Tea (KST3), Synergy 1 (S1KT4), Ninja Kombucha Cranberry Ginger Lime (NKT5), Ninja Kombucha Goldenberry (NKT6), Synergy 2 (S2KT7), Blue Ridge Bucha Elderberry Lime (B1KT8), Blue Ridge Bucha Ginger Hibiscus 1 (B2KT9), Blue Ridge Bucha Ginger Hibiscus 2 (B2KT10), Sage Mermaid Blueberry Pomegranate (MKT11), Sage Mermaid Ginger Lemon (MKT12), Family tea (TKT13), and blanks.

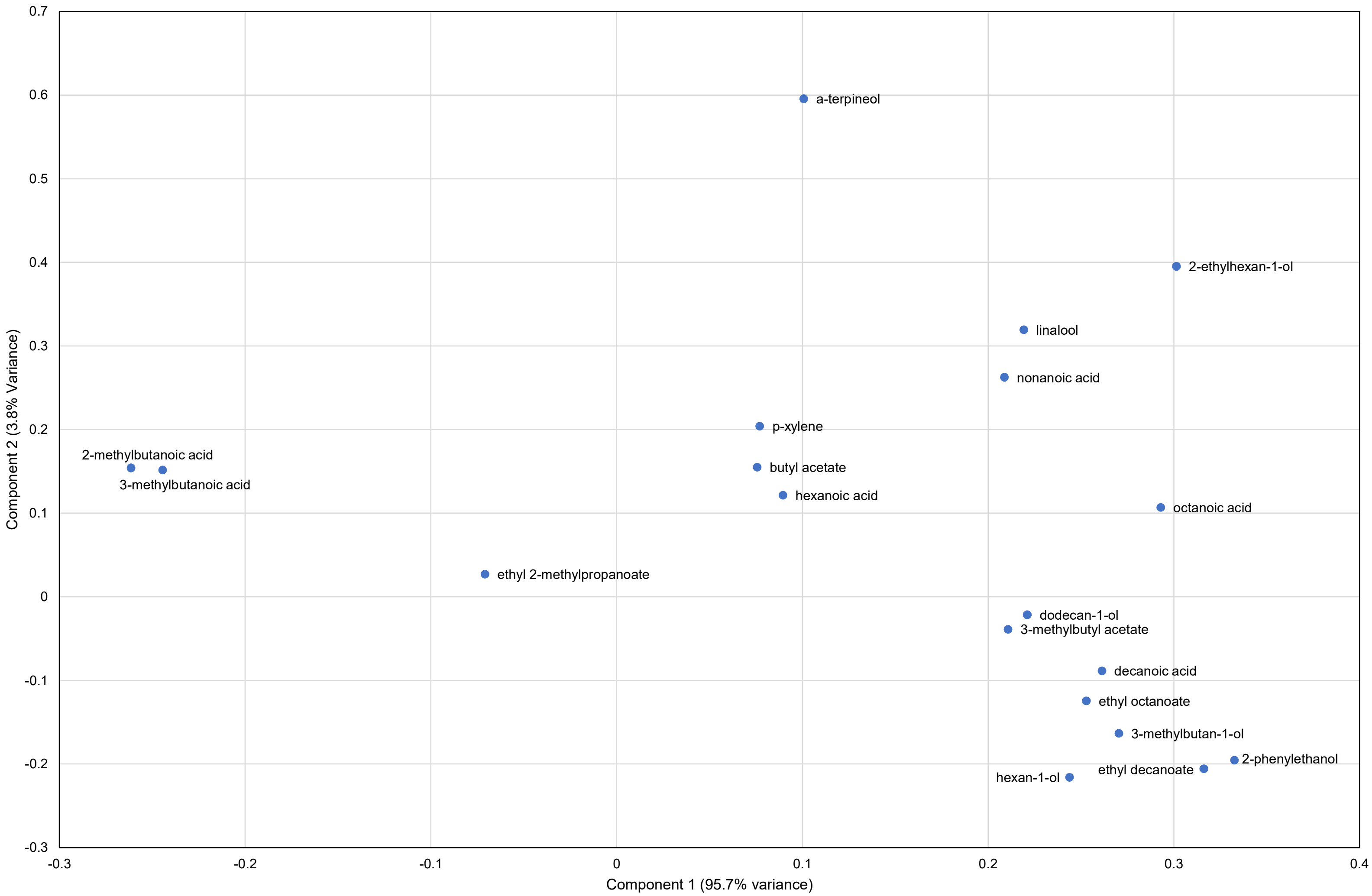

Figure S5. Principal Component Analysis (PCA) Loadings plot showing component 1 vs. component 2 for fold change analysis comparing SCOBY 1(SC1) to Family Kombucha (TKT13). Points to the left of the zero on the component 1 axis contributed to the grouping of SC1 and points to the right of the zero on the component 1 axis contributed to the grouping of the Family Kombucha . Loadings correspond to how the data is dispersed in the PCA Scores plot.
